# High-mobility semiconducting polymers with different spin ground states

**DOI:** 10.1038/s41467-022-29918-w

**Published:** 2022-04-26

**Authors:** Xiao-Xiang Chen, Jia-Tong Li, Yu-Hui Fang, Xin-Yu Deng, Xue-Qing Wang, Guangchao Liu, Yunfei Wang, Xiaodan Gu, Shang-Da Jiang, Ting Lei

**Affiliations:** 1 Key Laboratory of Polymer Chemistry and Physics of Ministry of Education, School of Materials Science and Engineering, Peking University, Beijing, 100871 China; 2 College of Chemistry and Molecular Engineering, Peking University, Beijing, 100871 China; 3 School of Polymer Science and Engineering, Center for Optoelectronic Materials and Devices, The University of Southern Mississippi, Hattiesburg, MS 39406 USA; 4 Spin-X Institute, School of Chemistry and Chemical Engineering, South China University of Technology, Guangzhou, China; 5 Beijing Key Laboratory for Magnetoelectric Materials and Devices, Peking University, Beijing, 100871 China

**Keywords:** Conjugated polymers, Electronic devices, Chemical physics

## Abstract

Organic semiconductors with high-spin ground states are fascinating because they could enable fundamental understanding on the spin-related phenomenon in light element and provide opportunities for organic magnetic and quantum materials. Although high-spin ground states have been observed in some quinoidal type small molecules or doped organic semiconductors, semiconducting polymers with high-spin at their neutral ground state are rarely reported. Here we report three high-mobility semiconducting polymers with different spin ground states. We show that polymer building blocks with small singlet-triplet energy gap (Δ*E*_S-T_) could enable small Δ*E*_S-T_ gap and increase the diradical character in copolymers. We demonstrate that the electronic structure, spin density, and solid-state interchain interactions in the high-spin polymers are crucial for their ground states. Polymers with a triplet ground state (*S* = 1) could exhibit doublet (*S* = 1/2) behavior due to different spin distributions and solid-state interchain spin-spin interactions. Besides, these polymers showed outstanding charge transport properties with high hole/electron mobilities and can be both n- and p-doped with superior conductivities. Our results demonstrate a rational approach to obtain high-mobility semiconducting polymers with different spin ground states.

## Abstract (web summary)

Semiconducting polymers with high-spin at their neutral ground state are rarely reported. Here the authors synthesize three semiconducting polymers with different spin ground states and high hole/electron mobility, by appropriate choice of the building blocks’ singlet-triplet energy gap, spin distributions and solid-state interchain interactions.

## Introduction

**Fig. 1:**
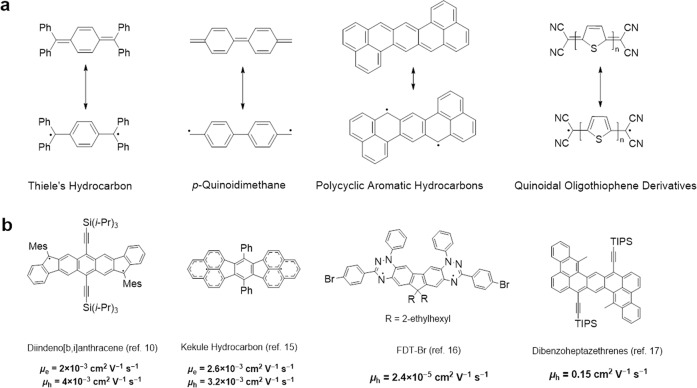
Previous works on the design and synthesis of open-shell or high-spin π-conjugated organic molecules. **a** Resonance structures of several open-shell π-conjugated organic molecules. **b** Some representative open-shell or high-spin organic semiconductors and their charge carrier mobilities.

π-Conjugated organic molecules with open-shell or high-spin ground state have attracted increasing interests due to their unique optoelectronic and magnetic properties^1–3^. However, these molecules contain unpaired electrons and are often unstable compared with the closed-shell molecules because they can easily form dimers, or being quenched due to their high reactivity. Recently, many efforts have been devoted to the development of stable open-shell conjugated molecules. Related strategies include the use of large steric hindrance groups to protect unpaired electrons, and delocalization of unpaired electrons to large conjugated systems^4^. Based on these strategies, some open-shell π-conjugated organic molecules, including *p*-quinodimethanes^5^, polycyclic aromatic hydrocarbons, quinoidal oligothiophene derivatives^6^ were investigated (Fig. 1a). Recent works on the design and precise synthesis of open-shell small molecules and oligomers have greatly expanded the open-shell small π-conjugated organic molecule library and provided a more in-depth understanding on their optoelectronic and magnetic properties^7–11^.

Charge carrier mobility is a critical parameter for an optoelectronic device, which reflects the speed at which the charge carriers move in a material. Open-shell organic molecules with high mobility will enable long spin transport distance which is crucial for spin-related applications^12–14^. However, there are few reports of small open-shell molecules with high charge carrier mobilities^10,15–17^. This is because the use of steric hindrance groups prevents close packing of molecules, and increasing the conjugation length requires more complex synthetic steps. Figure 1b shows some representative π-conjugated open-shell molecules, which usually show low charge carrier mobilities and only a few show mobilities over 0.1 cm^2^ V^−1^ s^−1^ ^17^. The low charge carrier mobilities of these molecules greatly restrict their applications in optoelectronic and spin-related devices.

Conjugated polymers have been intensively studied for various optoelectronic applications because of their high mechanical flexibility/stretchability and large-scale solution processability^18–20^. In the past decade, the development of high-performance polymer building blocks, including indacenodithiophene^19^, naphthalene diimide (NDI)^21^, diketopyrrolopyrrole (DPP)^22^, isoindigo (IID)^23^, and benzodifurandione-based oligo(p-phenylene vinylene) (BDOPV)^24^, have greatly promoted the charge carrier mobility enhancement in conjugated polymers. Conjugated polymers with open-shell character can be obtained by chemical doping^25^. However, the doped polymers are unstable and the ionized dopants always result in large structural and energetic disorders^26^. In addition, doped semiconducting polymers are always conductors while losing their semiconducting properties. Recently, conjugated polymers at their neutral ground state were reported to have open-shell high-spin character^27,28^, but these polymers showed conducting properties with limited applications in semiconducting devices. To date, the guidelines for the design of these polymers are rarely explored, and the influential factors for their spin properties are still obscure.

**Fig. 2:**
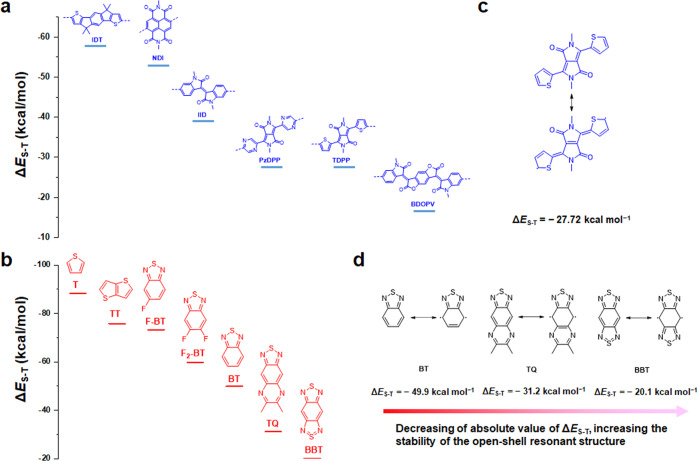
Computer-aided polymer building block screening approach to the rational design of high-spin ground-state semiconducting polymers. DFT Calculated Δ*E*_S-T_ values of **a** “large fused aromatic” and **b** “small-size aromatic” building blocks used in high-mobility semiconducting polymers; **c** The closed-shell and open-shell resonance structures of TDPP. **d** The closed-shell and open-shell resonance structures of BT, TQ, and BBT.

Here we report a strategy to design high-spin ground state and high-mobility semiconducting polymers. This approach is based on three assumptions: (i) Most high-mobility conjugated polymers are based on several high-performance building blocks (e.g., NDI, IID, and DPP etc. in Fig. 2a), and thus these building blocks are needed; (ii) The single-triplet energy gap (Δ*E*_S-T_ = *E*_S_−*E*_T_) is the energy difference between open-shell singlet state and open-shell triplet state^1^; as the singlet-triplet energy gap (Δ*E*_S-T_) value increase, the stability of the high-spin triplet state will increase, and the triplet state may become the ground state^5^; (iii) the polymers need to have a highly planar backbone, which can lead to better conjugation, smaller Δ*E*_S-T_, and also benefits charge transport. Based on these assumptions, we screened currently available high-performance polymer building blocks by calculating their Δ*E*_S-T_ values and planarity indexes. Three polymers were synthesized for comparison. These polymers exhibit distinct magnetic properties and solid-state spin-spin interactions. Among them, p(TDPP-TQ) and p(TDPP-BBT) exhibit an air-stable triplet ground state. Moreover, p(TDPP-TQ) displays high electron mobilities of up to 7.76 cm^2^ V^−1^ s^−1^ and high hole mobilities of up to 6.16 cm^2^ V^−1^ s^−1^. Interestingly, the polymer can also be effectively n-doped and p-doped, showing outstanding n-type and p-type electrical conductivities (*σ*_e_ = 16.1 S/cm and *σ*_h_ = 348.3 S/cm). We believe that our design strategy could help to discover more high-mobility semiconducting polymers with different spin ground states.

## Results

### Polymer design and synthesis

To date, many high-mobility semiconducting polymers are based on the copolymerization of “large fused aromatics” and “small-size aromatics”. Thus, we collected potential polymer building blocks from recently published reviews^22,29^ and separate them into two groups: “large fused aromatics” and “small-size aromatics” (Fig. 2a, b). Based on the above assumptions, we first performed DFT calculations to obtain their Δ*E*_S-T_ values. Among all the building blocks, TDPP, BDOPV, TQ, and BBT exhibit the smallest |Δ*E*_S-T_|. Then, we performed DFT calculations to estimate the planarity of the polymer building block combinations by using a recently developed planarity index 〈cos^2^*φ*〉^30^. We found that TDPP can form planar polymer backbone with TQ and BBT with high torsional barriers and large 〈cos^2^*φ*〉 (Fig. 1c). Whereas when BDOPV was copolymerized with TQ and BBT, the resulting polymers show small 〈cos^2^*φ*〉 values, suggesting the polymers significantly deviate from planarity (Supplementary Fig. 1). Note that the backbone planarity also strongly affects the Δ*E*_S-T_ values (Supplementary Table 1). Better backbone planarity could lead to smaller |Δ*E*_S-T_| values and more stable triplet states. Therefore, backbone planarity is crucial for both efficient intrachain and interchain charge transport, as well as for realizing high-spin ground states. To have a systematic understanding of the relationship between molecular structure and open-shell property, we choose TDPP as the “large fused aromatic”, and BT, TQ and BBT with decreased Δ*E*_S-T_ values, as the “small-size aromatic” to construct polymers. Note that TDPP, BT, TQ and BBT are usually considered as “acceptors” in conjugated polymers. Unlike traditional donor-acceptor design^27^, this “acceptor-acceptor” design allows the polymers to have enough bandgap to show semiconducting rather than conducting property.

**Fig. 3:**
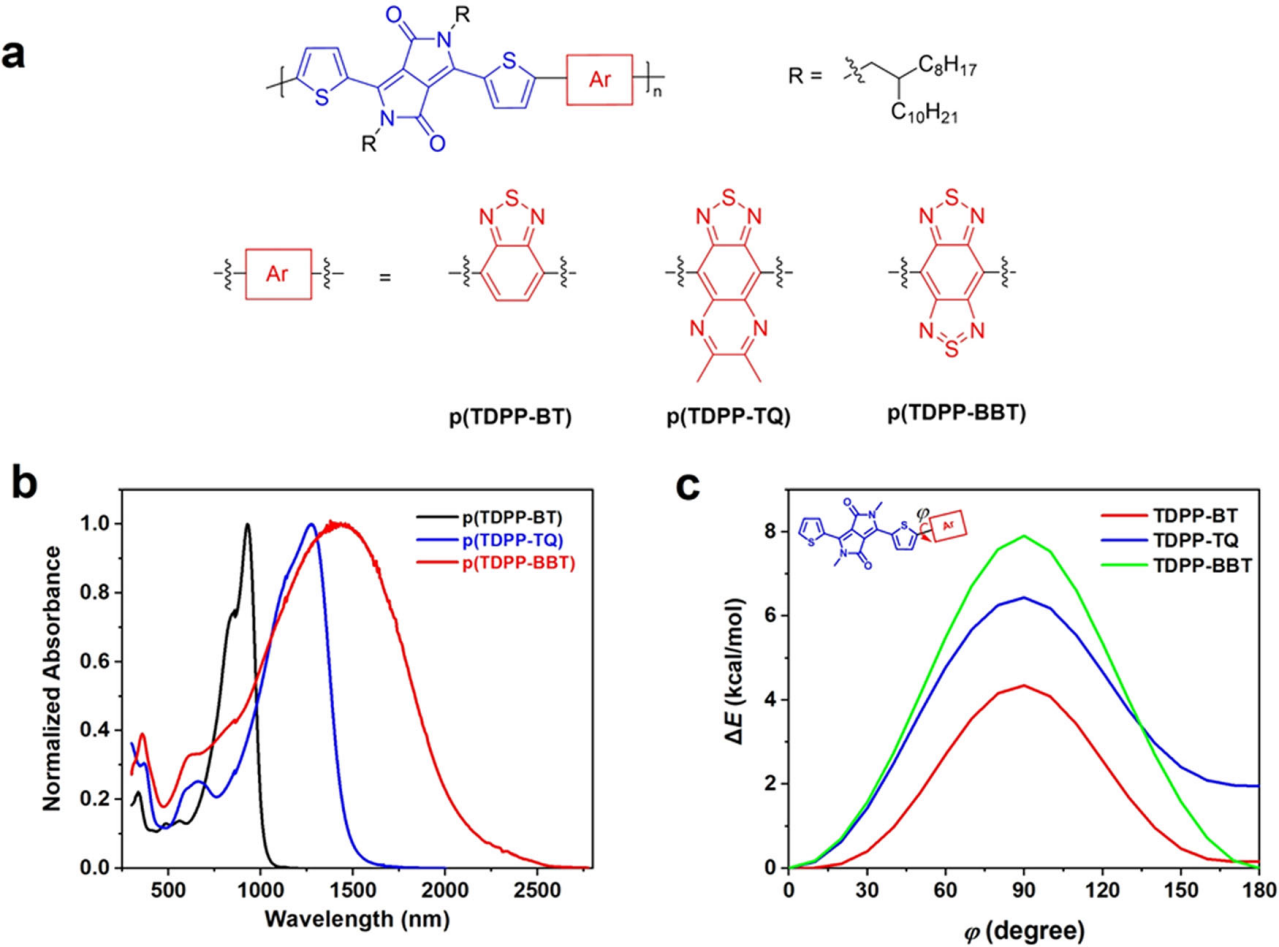
Chemical structure and characterization. **a** Molecular structures of three semiconducting polymers. “Ar” is the aromatic building block. **b** UV–vis–NIR absorption spectra of the polymers in ODCB solution (1 × 10^−5^ M). **c** Calculated potential energy scans (PES) of the dihedral angles *φ* between the TDPP and three BT derivatives.

The three polymers, p(TDPP-BT), p(TDPP-TQ), and p(TDPP-BBT), were synthesized via Pd-catalyzed Stille polymerization reaction between the trimethyltin TDPP and the dibromo compounds of BT, TQ, and BBT (Fig. 3a). The synthesis and purification procedures are detailed in Supplementary Materials. The stability of those polymers was proved by thermogravimetric analysis, and all the polymers showed high decomposition temperatures over 350 °C (Supplementary Fig. 2). Three polymers show gradually red-shifted absorption spectra as decreasing the Δ*E*_S-T_ of the “small-size aromatic” (Fig. 3b). The HOMO/LUMO energy levels of p(TDPP-BT), p(TDPP-TQ) and p(TDPP-BBT) obtained by cyclic voltammogram measurements are −5.34/−3.55, −5.23/−3.94, and −5.20/−4.17 eV, respectively (Supplementary Fig. 3). The increase of the HOMO and the decrease of the LUMO level is probably due to the enhanced planarity, better conjugation, and more readily aromatic-to-quinoidal transformation (Fig. 3c and Supplementary Fig. 3).

### Characterization of magnetic properties

**Fig. 4:**
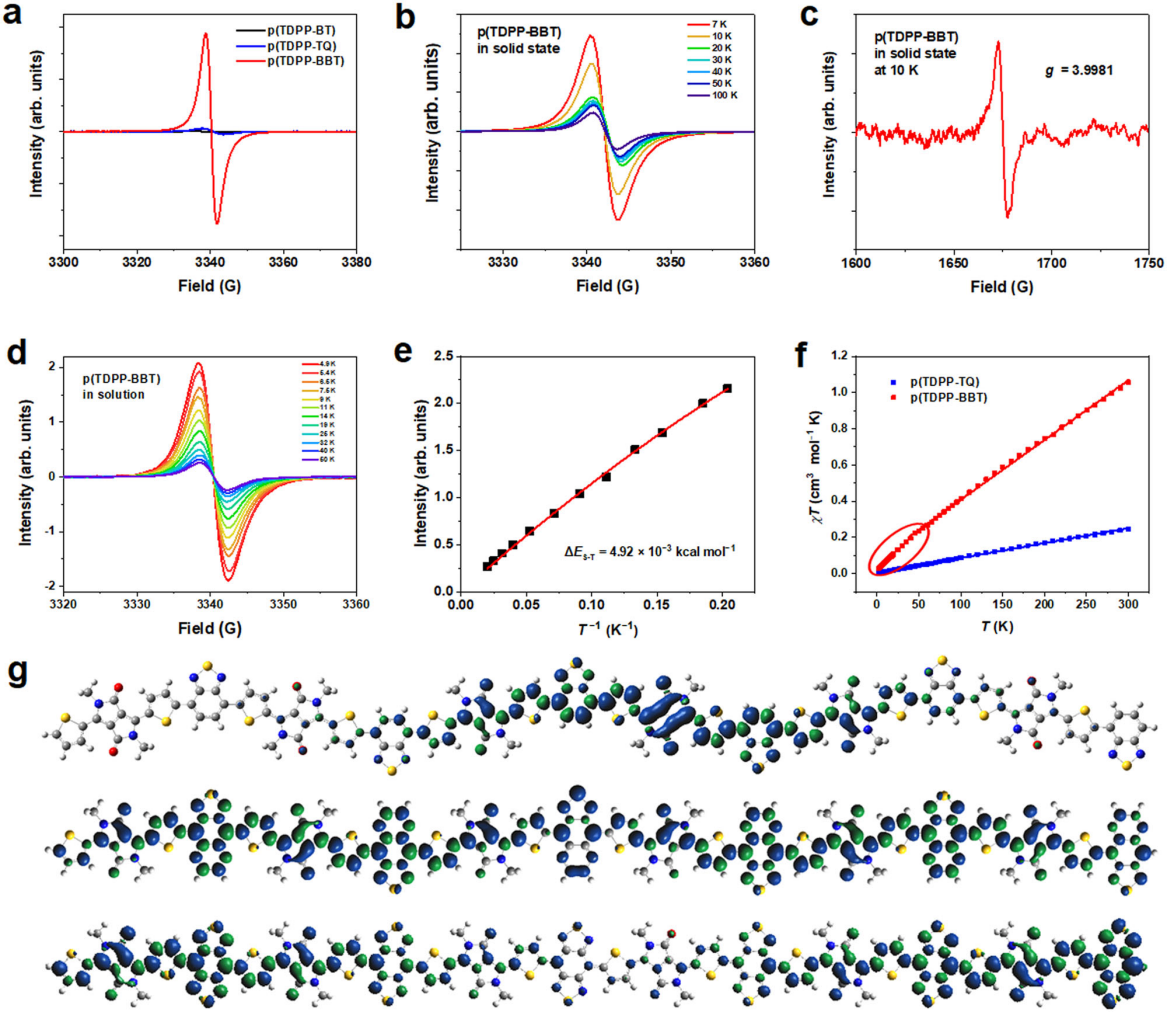
Magnetic property characterization and DFT calculation. **a** Room temperature EPR signals of the polymers in the solid state; **b** variable temperature EPR of p(TDPP-BBT) in the solid state; **c** the half field line of p(TDPP-BBT) in the solid state: |Δ*m*_s_| = 2 signal (*g* factor = 3.9981, the theoretical value: *g* = 4.0046); **d** temperature-dependent EPR of p(TDPP-BBT) in 1 × 10^−3^ M *o*-xylene and **e** the corresponding Bleaney-Bowers equation fitting result; **f** temperature-dependent magnetic susceptibility from 2 K to 300 K. Solid squares are data, solid lines are linear fitting lines. **g** spin density distribution of the triplet states of the oligomers (*n* = 6) of p(TDPP-BT) (top), p(TDPP-TQ) (middle), p(TDPP-BBT) (bottom). DFT calculations were performed at the UB3LYP/6-31 G** level. Atoms’ colors: gray for C, red for O, blue for N, yellow for S, and white for H.

The high-spin characteristics of the polymers were investigated by electron paramagnetic resonance (EPR) and superconducting quantum interference device (SQUID) both in solution and in the solid state. At room temperature, the solid-state EPR intensity increases dramatically in the order of p(TDPP-BT), p(TDPP-TQ), and p(TDPP-BBT) (Fig. 4a), which reflects an increase of spin density. More importantly, the EPR intensities showed negligible change after storing the polymers in air for 90 days, suggesting the high air stability of these open-shell polymers (Supplementary Fig. 4). The EPR intensity of p(TDPP-BT) is very weak and shows a close-shell feature, and thus we will not study this polymer in detail. To compare the spin dynamics of p(TDPP-TQ) and p(TDPP-BBT), temperature-dependent EPR measurements in the solid state were performed (Fig. 4b, Supplementary Fig. 5a). Their EPR intensities decrease with the increase of temperature, suggesting that they probably have a triplet ground state^1^. It is known that if there exists a triplet state ground state, the EPR signal of |Δ*m*_s_| = 2 forbidden transition can be observed, even with very weak intensity^1^. To determine the existence of triplet ground state in the polymers, the |Δ*m*_s_| = 2 forbidden transition was measured. For p(TDPP-BBT), a low intensity but clear |Δ*m*_s_| = 2 signal (*g* factor = 3.9981) is observed (Fig. 4c), suggesting the existence of triplet ground state. For p(TDPP-TQ), the forbidden transition could not be observed, which is largely due to its relatively weak EPR intensity compared to p(TDPP-BBT). Furthermore, the variable temperature EPR of both polymers in solution was also measured and showed a similar behavior compared to solid state EPR. The EPR intensities of p(TDPP-TQ) and p(TDPP-BBT) decrease with the increase of temperature (Fig. 4d, Supplementary Fig. 5b). The EPR data of p(TDPP-BBT) from 4.9 K to 50 K were fitted by the Bleaney-Bowers equation, which provides an energy gap between singlet and triplet (Δ*E*_S-T_) of 4.92 × 10^−3^ kcal mol^−1^ (*J* = 2.48 K) (Fig. 4e).

To further investigate the static magnetic properties of the polymers, temperature-dependent magnetic susceptibility was measured by SQUID from 2 K to 300 K under DC field (Fig. 4f). The values of the product of magnetic susceptibility and temperature (*χ*_M_*T*) increase linearly with temperature. This feature usually suggests strong antiferromagnet coupling or temperature-independent paramagnetism (TIP). Due to the relatively low calculated spin density and weak spin-orbit coupling in organic polymers, this phenomenon is usually attributed to TIP^31,32^. TIP comes from Curie and Pauli paramagnetism (*χ*_total_*T* = C + *χ*_Pauli_*T*), where *C* is Curie paramagnetism and *T* is temperature. After fitted the *χT*–*T* plot, the *χ*_Pauli_ of p(TDPP-TQ) and p(TDPP-BBT) were determined to be 8.2 × 10^−4^ and 3.5 × 10^−3^ cm^3^ mol^−1^, and Curie constants are 3.2 × 10^−3^ and 4.0 × 10^−2^ cm^3^ mol^−1^ K, respectively. From the relation *χ*_Pauli_ = *μ*_B_^2^*N*(*E*_F_), where *μ*_B_ is the Bohr magneton, the densities of states at the Fermi energy *N*(*E*_F_) for those p(TDPP-TQ) and p(TDPP-BBT) were calculated to be 1.5 × 10^22^ and 6.5 × 10^22^ eV^−1^ cm^−3^. The Pauli paramagnetism and large value of *N*(*E*_F_) reveal that the spins in these polymers are highly delocalized^32,33^ (see detailed discussions in the Supplementary Information Supplementary Note 4). In particular, for p(TDPP-BBT), the *χ*_M_*T* and *T* are slightly deviated from the linear relationship at low temperature (red circle in Fig. 4f). This behavior could be due to the weak antiferromagnetic coupling between polymer chains, which we will prove later by studying the aggregation behavior of the polymers and the DFT calculations of oligomers and dimers.

### Understanding of the different spin ground states

**Fig. 5:**
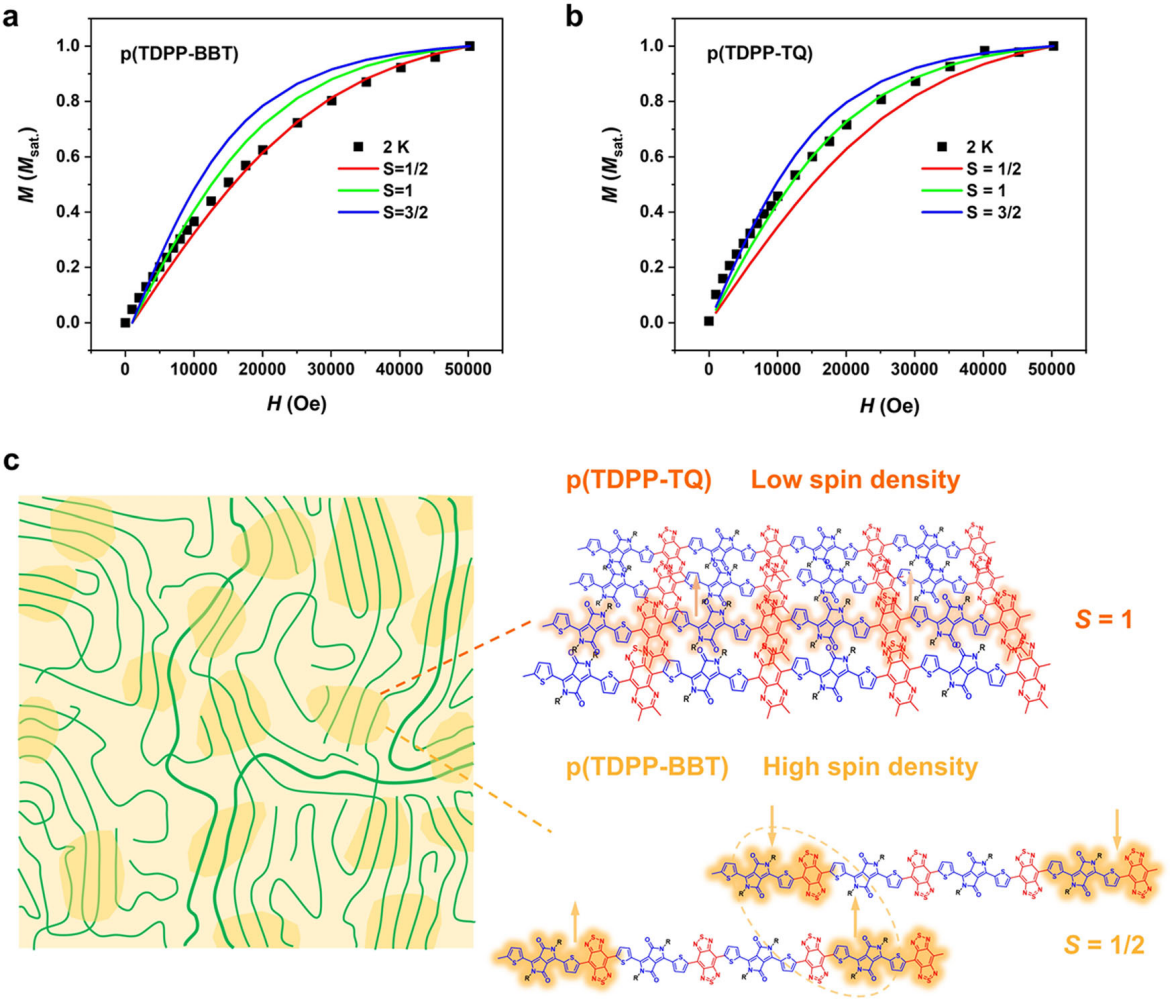
Magnetic characterization and the mechanism explanation of the different spin ground states of high-spin polymers. Field dependent magnetization measurement of **a** p(TDPP-TQ) and **b** p(TDPP-BBT) from 0 Oe to 50,000 Oe. The solid squares are the experimental data, the solid lines stand for the simulated curve by Brillouin equation. The simulation results exhibit the S = 1 and S = 1/2 ground states of p(TDPP-TQ) and p(TDPP-BBT). **c** Schematic illustration of the mechanism of the different ground states of p(TDPP-TQ) and p(TDPP-BBT). Left image shows the microstructure of a typical polymer film, which contains ordered crystalline regions (highlighted in darker yellow) and amorphous regions. Right image illustrates the spin-spin interactions in solid state. The orange shadings on the polymers indicate where the spins mostly distribute. In p(TDPP-TQ), the low spin density makes the spins mostly interact within a chain. While in p(TDPP-BBT), we observed interchain spin-spin interactions because of its high spin density.

To determinate the spin ground state of those polymers, field-dependent magnetization measurements (*M*–*H*) were performed. For p(TDPP- TQ), it shows a close to *S* = 1 triplet state (Fig. 5a), consistent with EPR results; while for p(TDPP-BBT), it shows a close to *S* = 1/2 doublet state in the measurement (Fig. 5b). It seems that this result conflicts with the EPR measurement. However, we will show that this phenomenon can be well explained as following.

First, p(TDPP-BBT) has a long polymer chain (~20 repeat units for one chain) with spins distributed at the chain ends. We performed density functional theory (DFT) calculations for their oligomers (*n* = 6). The calculated Δ*E*_S-T_ of the oligomers were −19.50 kcal mol^−1^ for p(TDPP-BT), −3.69 kcal mol^−1^ for p(TDPP-TQ), and −0.14 kcal mol^−1^ for p(TDPP-BBT), which is consistent with the above observed trend that p(TDPP-BBT) could have very small Δ*E*_S-T_ value and a triplet ground state. The significant decrease of |Δ*E*_S-T_| indicates the increased stability of the open-shell triplet ground state, which agrees well with the observed EPR results and magnetic property. The large |Δ*E*_S-T_| value of p(TDPP-BT) leads to the inaccessibility of the triplet state even at room temperature. While for p(TDPP-TQ) and p(TDPP-BBT), the small |Δ*E*_S-T_| value of their oligomers could make the polymers have triplet ground state if the polymers have high degree of polymerization and strong interchain interactions. The singlet diradical character index (*y*_0_), which is often used to estimate the diradical property, was also calculated^34,35^. The value of *y*_0_ ranges from 0 to 1; 0 means closed-shell, while *y*_0_ = 1 stands for pure diradical. The calculated *y*_0_ values of (TDPP-BT)_6_, (TDPP-TQ)_6_, and (TDPP-BBT)_6_ are 0.012, 0.350, and 0.996 (Supplementary Tables 4–6). The difference of the spin triplet states for each polymer can be visualized by the spin density distribution in Fig. 4g. From (TDPP-BT)_6_ to (TDPP-BBT)_6_, the triplet spin density distribution changes from localized in the center, to uniformly distributed along the chain, and to mostly distributed at the end of the chain. This result agrees well with the increase of the *y*_0_ value (Supplementary Figs. 14–16). The bond length alternation analysis (Supplementary Figs. 17, 19) also shows that the bond length difference between open-shell singlet and triplet of (TDPP-BT)_6_ become obvious only in the middle of the molecule. For (TDPP-TQ)_6_, it emerges throughout the entire oligomer. For (TDPP-BBT)_6_, the bond length difference is negligible, which is consistent with its small |Δ*E*_S-T_| value. It has been proposed that when the two spins are separated at a long distance, the two spins have weak interactions and can be regarded as two radicals (or diradical)^1^. Similar results have been observed in a quinoidal oligomer system^36^. They found that when oligomer number ≥4, the triplet state of the oligomer is better to be described by two individual radicals (also known as biradicals).

Second, the strong interchain interactions between p(TDPP-BBT) chains might also lead to the doublet ground state. It has been reported that conjugated polymers with rigid backbones are strongly aggregated even in dilute solutions^37,38^. The strong intermolecular interaction will affect spin delocalization^31^. To understand the polymer chain interactions, we performed the temperature-dependent UV–vis absorption spectra of p(TDPP-TQ) and p(TDPP-BBT) (Supplementary Figs. 10 and 11). We and others have shown that in good solvent (dissolving polymers better), conjugated polymers can be more readily disaggregated at elevated temperature^37,39^. Therefore, three solvents, toluene, *o*-dichlorobenzene (*o*-DCB), and 1-chloronaphthalene (1-CN), with increased solubility for conjugated polymers were used. For both polymers, the maximum absorption peak decreases with increasing temperature and decreases more significantly in a good solvent, such as 1-CN, suggesting that both polymers are strongly aggregated in solution. The strong aggregation of the polymer supports the observed large *N*(*E*_F_) values, which could explain the TIP phenomenon. Previous study has shown that strong spin-spin interactions in triplet small molecules could result in doublet state in magnetization measurements^15^. p(TDPP-TQ) has a low spin density and direct spin-spin interaction can hardly happen (Fig. 5c), thus exhibiting a triplet ground state in solid state. According to the quantitative ESR measurement (Supplementary Fig. 6), we can obtain the number of spins per polymer chain is 0.98, close to 1, at room temperature for p(TDPP-BBT). The high spin density makes the spin at the chain end can directly interact with each other, leading to an apparent doublet ground state, consistent with the field-dependent magnetization measurement.

To support this hypothesis, we also performed DFT calculations for the polymer dimers of the *n* = 4 oligomer to compare the energy, molecular orbitals, and spin density between a single polymer chain and dimers with different interchain packings (Supplementary Figs. 20, 23). As expected, all the stacked dimers of three polymers are more stable than two single chains, indicating the strong π-π stacking interactions between the polymer chains (Supplementary Fig. 21). p(TDPP-BT) dimers showed a large negative Δ*E*_S-T_ value, making it hard to access the triplet state. This is consistent with our result that p(TDPP-BT) is a close-shell polymer with a singlet ground state. We then focus on the difference between p(TDPP-BBT) and p(TDPP-TQ). As shown in Supplementary Fig. 22, the frontier orbitals of p(TDPP-BBT) and p(TDPP-TQ) dimers have shown obvious overlaps between two chains, suggesting the two polymer chains have strong electronic couplings^40^. Spin density redistribution is observed for both p(TDPP-TQ) and p(TDPP-BBT), indicating the existence of strong intermolecular spin-spin interaction (Supplementary Fig. 23). Interestingly, the two polymers exhibit significantly different spin distributions. The intermolecular electron coupling through π-π stacking decreases the spin density in the middle of the p(TDPP-BBT) dimer. This feature increases the distance of the two unpaired spins located at the end of the dimer and diminishes the electron exchange interaction, leading to the formation of two independent radicals (doublet state). However, for p(TDPP-TQ), the spin is distributed all along the polymer chain for both the single chain and the dimer. Thus, for p(TDPP-TQ), the two radicals are closer with stronger interactions, forming diradicals and exhibiting triplet ground state in solid-state.

Based on the magnetic properties and DFT calculations, the following conclusion can be obtained: (1) p(TDPP-BT) has a singlet ground state because of its large Δ*E*_S-T_ value; (2) p(TDPP-TQ) and p(TDPP-BBT) have triplet ground state because of their intrinsically small Δ*E*_S-T_ values; (3) because of high spin density, separately distributed spin-density, and interchain spin-spin interactions in p(TDPP-BBT), it shows doublet state in solid state; whereas the low spin density and relatively uniformly distributed spin density in p(TDPP-TQ) make it exhibit triplet ground state.

### Charge-transport properties and thin-film characterization

**Fig. 6:**
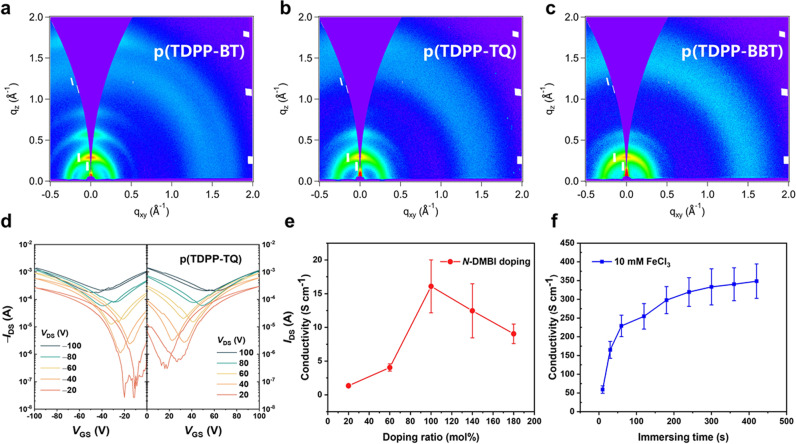
Thin film morphology and device characterization. 2D-grazing incidence wide-angle X-ray scattering (GIWAXS) pattern of **a** p(TDPP-BT), **b** p(TDPP-TQ), **c** p(TDPP-BBT). **d** Typical transfer characteristics of a p(TDPP-TQ) FET device. **e** N-type electrical conductivities of p(TDPP-TQ) doped by *N*-DMBI with different ratios; **f** P-type electrical conductivities of p(TDPP-TQ) doped by immersing in 10 mM FeCl_3_ solution. Error bars indicate the standard deviation of ten experimental replicates.

The charge transport properties of the polymers were evaluated by field-effect transistors with a top-gate/bottom-contact configuration (Fig. 6d, Supplementary Figs. 24, 27). Unlike previously reported high-spin polymers with conducting properties^27^, our polymers show typical and close to ideal semiconducting properties with good ambipolar charge transport properties. The polymers also showed good on/off ratios if the *V*_DS_ is small. p(TDPP-BT) exhibits good electron mobilities of up to 3.83 ± 0.65 cm^2^ V^−1^ s^−1^ and hole mobilities of up to 2.77 ± 0.15 cm^2^ V^−1^ s^−1^ in the saturation regime. p(TDPP-TQ) exhibits high electron mobilities of up to 7.76 ± $0$.86 cm^2^ V^−1^ s^−1^ and high hole mobilities of up to 6.16 ± $0$.68 cm^2^ V^−1^ s^−1^ in the saturation regime. These values are the highest reported to date in high-spin ground state polymers, and also among the highest in all reported organic semiconductors^22^. p(TDPP-BBT) shows relatively lower electron mobilities of 0.35 ± 0.08 cm^2^ V^−1^ s^−1^ and hole mobilities of 0.25 ± 0.06 cm^2^ V^−1^ s^−1^. Their charge carrier mobilities in the linear regime are also calculated (Supplementary Table 7). To date, only a few reported open-shell small molecules have shown moderate charge carrier mobilities, usually on the order of 10^−3^ cm^2^ V^−1^ s^−1^ (Fig. 1b and Supplementary Table 8)^10,15,16,41,42^. Therefore, our approach successfully addressed the challenge to design high-mobility and high-spin ground-state organic semiconductors.

Grazing incidence wide-angle X-ray scattering (GIWAXS) and atomic force microscopy (AFM) were employed to study the microstructures and morphology of the polymer films. (Fig. 6a–c, Supplementary Fig. 30). All the polymers show typical face-on dominated molecular packing in solid state. p(TDPP-BT) shows the narrower full width at half maximum (FWHM) (Supplementary Fig. 32 and Supplementary Table 9) with a lamellar distance of 21.67 Å and a π–π stacking distance of 3.57 Å, suggesting its higher ordered crystallite domain. p(TDPP-TQ) shows wider FHWM with a lamellar distance of 20.94 Å and a π–π stacking distance of 3.70 Å. For p(TDPP-BBT) (Fig. 6c), it shows the widest FWHM and indistinguishable π–π stacking peak. After re-measuring its film at Shanghai Synchrotron Radiation Facility (Supplementary Fig. 33), we finally identified that p(TDPP-BBT) has a lamellar distance of 20.27 Å and a π–π stacking distance of 3.69 Å. The poor crystallinity of p(TDPP-BBT) might explain its relatively lower charge carrier mobilities. AFM height images show that all the polymer films have a very smooth surface with root-mean-square surface roughness <1 nm (Supplementary Fig. 30). Note that the charge transport properties of the three polymers are not strongly correlated with their molecular packings. This phenomenon is common in conjugated polymers, because many studies have shown that crystallinity and π–π stacking distance of polymers are not the only parameters affecting their charge transport properties, and other parameters, such as interchain short contacts^43^, packing conformation^44^, and energetic disorders^45^, also strongly influences charge carrier mobilities.

Previous studies suggest that spin-spin interactions in materials could lead to enhanced thermopower in some thermoelectric materials^46,47^. Since organic thermoelectric materials are usually achieved under doped states, we explored the charge transport properties after doping. We found that p(TDPP-TQ) can be both effectively n-doped and p-doped. For n-doping, a commonly used n-dopant, *N-*DMBI, was used. After optimizing the doping concentration and annealing temperature, the n-type electrical conductivity of p(TDPP-TQ) polymer achieved 16.1 S/cm (Fig. 6e), which is among the highest in n-doped organic semiconducting polymers^48,49^. P-doping was performed by immersing p(TDPP-TQ) films in a 10 mM FeCl_3_ solution. The polymer can be easily p-doped as observed in the UV−vis−NIR absorption spectra (Supplementary Fig. 31). By varying the immersion time, the polymer showed a p-type electrical conductivity of 348.3 S/cm (Fig. 6f), which is also among the highest in p-doped conjugated polymers. One polymer that can achieve both high n-type conductivity and p-type conductivity after doping is rare in literature^50,51^. Compared with other polymers, both n- and p-doped p(TDPP-TQ) polymer films exhibited mediate stability in the ambient conditions (Supplementary Fig. 34). Such unique doping behavior of p(TDPP-TQ) could be probably due to the ease of accepting and donating electrons of the triplet state. These results also suggest the great potential of using high-spin semiconducting polymers in the applications requiring heavy doping, e.g., organic thermoelectrics and organic bioelectronics^50,52,53^.

## Discussion

In summary, we have developed a rational strategy to screen the potential polymer building blocks for designing high-spin ground state and high mobility semiconducting polymers. Based on the strategy, three polymers with different spin ground states were obtained. We found that the spin distributions and interchain interactions could lead to different spin behaviors for these polymers. p(TDPP-BT) has a singlet ground state, p(TDPP-TQ) has a triplet ground state, while p(TDPP-BBT) has a doublet ground state. More importantly, all these polymers showed high charge carrier mobilities among all the reported high-spin organic semiconductors, and they could also be readily p- and n-doped to show high electrical conductivities, suggesting their broad applications for versatile organic magnetic and electronic devices. We believe that the molecular design strategy developed in this work could bring more air-stable, high-mobility semiconducting polymers with different spin ground states, which could enable broad spin-related applications beyond the current scope of organic electronics.

## Methods

### Polymerization methods

The distannyl TDPP (0.05 mmol), dibromo BT or TQ or BBT (0.05 mmol), Pd(PPh_3_)_4_ (8 mol%, 4 μmol), and 0.2 mL xylene were put into a 0.5 mL microwave reaction tube. The reaction was drove by microwave with the following procedure: 120 °C for 5 min, 140 °C for 5 min, 170 °C for 30 min. A deep blue solution was formed after the reaction. After the reaction was finished, *N,N’*-diethylphenylazothioformamide (0.01 mmol) was added and stirred at 90 °C for 30 min to remove palladium or other residue metals. The mixture was poured to 100 mL methanol to precipitate the polymers and then filtered. The solid product was put into a soxhlet extraction and extracted by methanol (4 h), acetone (4 h), hexane (10 h), tetrahydrofuran (5 h), dichloromethane (5 h), chloroform (12 h) and finally collected by chlorobenzene (24 h). ^1^H NMR of three polymers is shown in Supporting Information.

### Electron paramagnetic resonance spectroscopy

Electron paramagnetic resonance (EPR) spectroscopy was conducted on a Bruker E580 spectrometer using ER 4122 SHQE highly sensitive EPR cavity. Oxford EPR900 cryostat was used for temperature control. The microwave frequency is at 9.37 GHz.

### SQUID measurement

The static magnetic property measurement was performed on a Quantum Design MPMS XL SQUID magnetometer from 2 to 300 K. The magnetization measurements were performed at 2 K over the magnetic field range of 250–70,000 Oe. The sample was packeted by aluminum foil and placed in a nonmagnetic holder. The diamagnetic contribution of the sample holder is negligible. The diamagnetic contribution of aluminum foil was corrected with blank holders. The diamagnetic contribution of the polymer was corrected by the Pascal’s constants.

## Supplementary information

Supplementary Information

## Data Availability

The source data generated in this study have been deposited in the materials cloud database (https://archive.materialscloud.org/record/2022.46) and are also available from the corresponding author upon request.

## Code availability

The codes that support the findings of this study are available from the corresponding authors upon request.
