# Supplementary material for: High-mobility semiconducting polymers with different spin ground states
Source: Nat Commun. 2022 Apr 26;13:2258. doi: 10.1038/s41467-022-29918-w (PMC9042904; doi:10.1038/s41467-022-29918-w)
Supplement: Supplementary file 1 — Supplementary Information [file 41467_2022_29918_MOESM1_ESM.pdf]

# Supplementary Information

*for*

# High-mobility semiconducting polymers with different spin ground states

Xiao-Xiang Chen[#], Jia-Tong Li[#], Yu-Hui Fang, Xin-Yu Deng, Xue-Qing Wang,

Guangchao Liu, Yunfei Wang, Xiaodan Gu, Shang-Da Jiang, Ting Lei[*]

Correspondence to T.L. tinglei@pku.edu.cn

**This PDF file includes:**

Supplementary Note 1. General procedures and experimental details

Supplementary Note 2. DFT calculations for the evaluation of polymer building block planarity

Supplementary Note 3. The thermal stability, cyclic voltammograms, and inductively coupled plasma (ICP) emission spectroscopy for the trace metal analysis of the polymers

Supplementary Note 4. EPR and SQUID measurement

Supplementary Note 5. Temperature-dependent UV-vis absorption spectra of p(TDPP-TQ) and p(TDPP-BBT)

Supplementary Note 6. DFT calculations of oligomers and dimers

Supplementary Note 7. Device fabrication and characterization

Supplementary Note 8. Material synthesis and characterization

Supplementary References

**Supplementary methods and discussions**

**Supplementary Note 1. General procedures and experimental details**

**Chemical reagents and characterization methods.** All chemical reagents were purchased and used as received unless otherwise indicated. All air and water sensitive reactions were performed under nitrogen atmosphere. Dichloromethane, tetrahydrofuran, toluene and *N, N*-Dimethylformamide were dried by a JC Meyer solvent drying system prior to use. $^{1}H$ NMR and $^{13}C$ NMR spectra were recorded on Bruker ARX-400 (400 MHz). All chemical shifts were reported in parts per million (ppm). $^{1}H$ NMR chemical shifts were referenced to $CDCl_3$ (7.26 ppm) and $^{13}C$ NMR chemical shifts were referenced to $CDCl_3$ (77.16 ppm). Mass spectra were recorded on an AB Sciex-5800 MALDI-TOF mass spectrometer and a Bruker Solarix XR mass spectrometer. Elemental analyses were performed on Vario EL elemental analyzer. Molecular weights of the polymers were determined by gel permeation chromatography (GPC) performed on Polymer Laboratories PL-GPC220 at 150 °C using 1,2,4-tricholorobenzene (TCB) as eluent. Thermal gravity analyses (TGA) were carried out on a TA Instrument Q600 SDT analyzer, and differential scanning calorimetry (DSC) analyses were performed on a TA Instrument Q2000 analyzer.

**Absorption spectra and cyclic voltammetry.** UV-vis-NIR absorption spectra and temperature-depending absorption spectra were performed on PerkinElmer Lambda 750 UV-vis spectrometer. Cyclic voltammetry (CV) was performed on BioLogic SP-300 workstation. Thin film measurements were carried out in an acetonitrile containing 0.1 M *n*-$Bu_4NPF6$ as a supporting electrolyte. Glassy carbon electrode was used as a working electrode and a platinum wire as a counter electrode, and all potentials were recorded versus AgCl/Ag (saturated) as the reference electrode (scan rate: 50 mV $s^{-1}$).

**Inductively coupled plasma (ICP) emission spectroscopy**. Polymers p(TDPP-BT) (10.2 mg), p(TDPP-TQ) (12.0 mg), p(TDPP-BBT) (9.9 mg) were heated to 600 °C for

10 h, and then the residue was digested in aqua regia for 10 h. The analysis was performed on Prodigy 7 ICP-atomic emission spectrometer.

**Atomic force microscopy (AFM) and GIWAXS measurements.** AFM spectropies were performed with a Cypher atomic force microscope (Asylum Research, Oxford Instruments). The surface morphology was recorded with a scan rate of 2-3 Hz at AC mode. GIWAXS experiment was performed on Xenocs Xuess 2.0 beamline, with an incident X-ray angle of 0.2 degrees and wavelength of 1.54 angstrom. The scattered signal was collected by Pilatus 1M detector at a sample to detector distance of 150 mm. Data processing was performed in Igor Pro software with Nika and WAXTools package.

**Supplementary Note 2. DFT calculations for the evaluation of polymer building block planarity.**

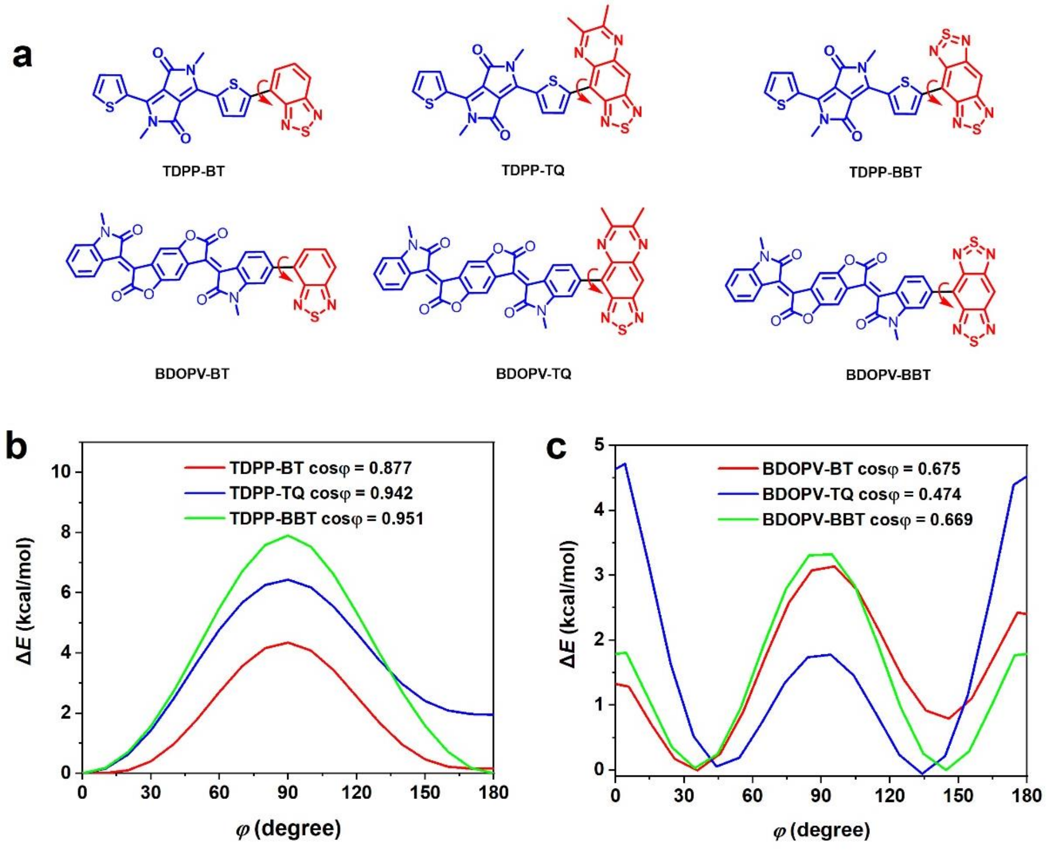


**Supplementary Figure 1.** Relaxed potential energy scans (PES) of the torsion angles $\varphi$ in TDPP-X and BDOPV-X. **a,** Molecule structure of TDPP-BT, TDPP-TQ, TDPP-

BBT, BDOPV-BT, BDOPV-TQ and BDOPV-BBT. The torsion angles $\varphi$ are the dihedral angles between the "large fused aromatics" and "small-size aromatics" segments. We employed an index $\langle \cos^2\varphi \rangle$ to quantify the planarity of molecules by considering all torsional conformations and their relative contribution to the overall structural disorder.[1] **b,** All the TDPP-based polymers exhibit dominant conformations with minimal energy at 0° or/and 180°, which could be attributed to the non-covalent interactions between hydrogen in thiphone and nitrogen in "small-size aromatics". TDPP-BT has the smallest $\langle \cos^2\varphi \rangle$ of 0.877 among these TDPP-copolymers due to its lowest energy barrier at 90°. As for BDOPV family (Fig.S1c) with much lower $\langle \cos^2\varphi \rangle$, i.e. much poorer planarity, the energy minimums are at around 40° and 130°, indicating large steric repulsion between the aromatic building blocks and thus large rotational disorder along the conjugated backbones.

**Supplementary Table 1.** DFT calculated $\Delta E_{S\text{-}T}$ values of the n = 1 oligomers of the TDPP and BDOPV polymers.

| Oligomer | $\Delta E_{S\text{-}T}$ (kcal mol$^{-1}$) |
|---|---|
| TDPP-BT | −20.50 |
| TDPP-TQ | −13.56 |
| TDPP-BBT | −7.60 |
| BDOPV-BT | −22.51 |
| BDOPV-TQ | −22.18 |
| BDOPV-BBT | −15.70 |

To evaluate the importance of backbone planarity to $\Delta E_{S\text{-}T}$, the $\Delta E_{S\text{-}T}$ values of TDPP-BT, TDPP-TQ, TDPP-BBT, BDOPV-BT, BDOPV-TQ, BDOPV-BBT were calculated (Supplementary Table 1). Even BDOPV has a less negative $\Delta E_{S\text{-}T}$ (−16.98 kcal mol$^{-1}$) value than TDPP (−27.72 kcal mol$^{-1}$), suggesting the triplet state of BDOPV is more stable. However, when BDOPV is polymerized with BT, TQ, BBT units, the $\Delta E_{S\text{-}T}$ values of the BDOPV-X (X = BT, TQ, BBT) become more negative than that of the corresponding TDPP-X (X = BT, TQ, BBT) (n = 1 oligomer). The reason is that TDPP-X oligomers have a better backbone planarity with close to 0° torsional angles, while BDOPV-X oligomers have torsion angles $\varphi$ around 40°.

**Supplementary Note 3. The thermal stability, cyclic voltammograms, and inductively coupled plasma (ICP) emission spectroscopy for the trace metal analysis of the polymers**

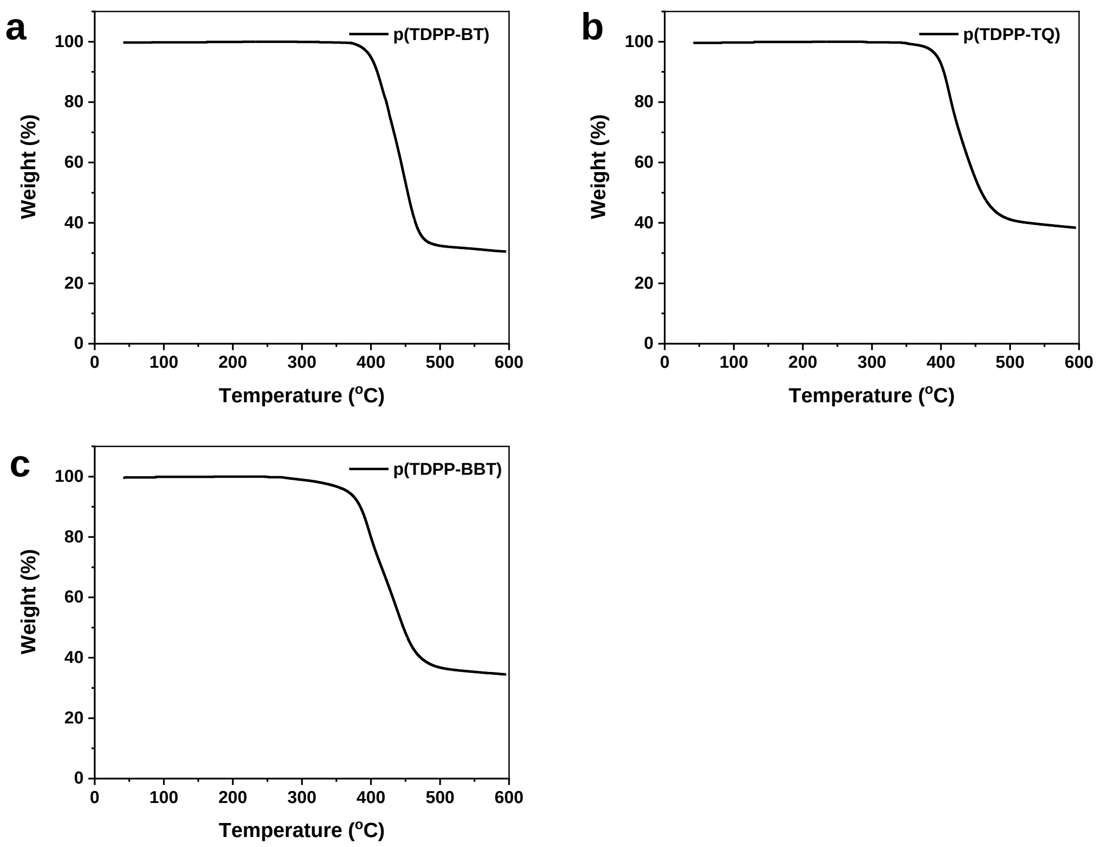


**Supplementary Figure 2.** Thermogravimetric analysis (TGA) of **a,** p(TDPP-BT), **b,** p(TDPP-TQ) and **c,** p(TDPP-BBT). The decomposition temperatures (5% weight loss) of three polymers are 400 °C, 393 °C, 365 °C, respectively.

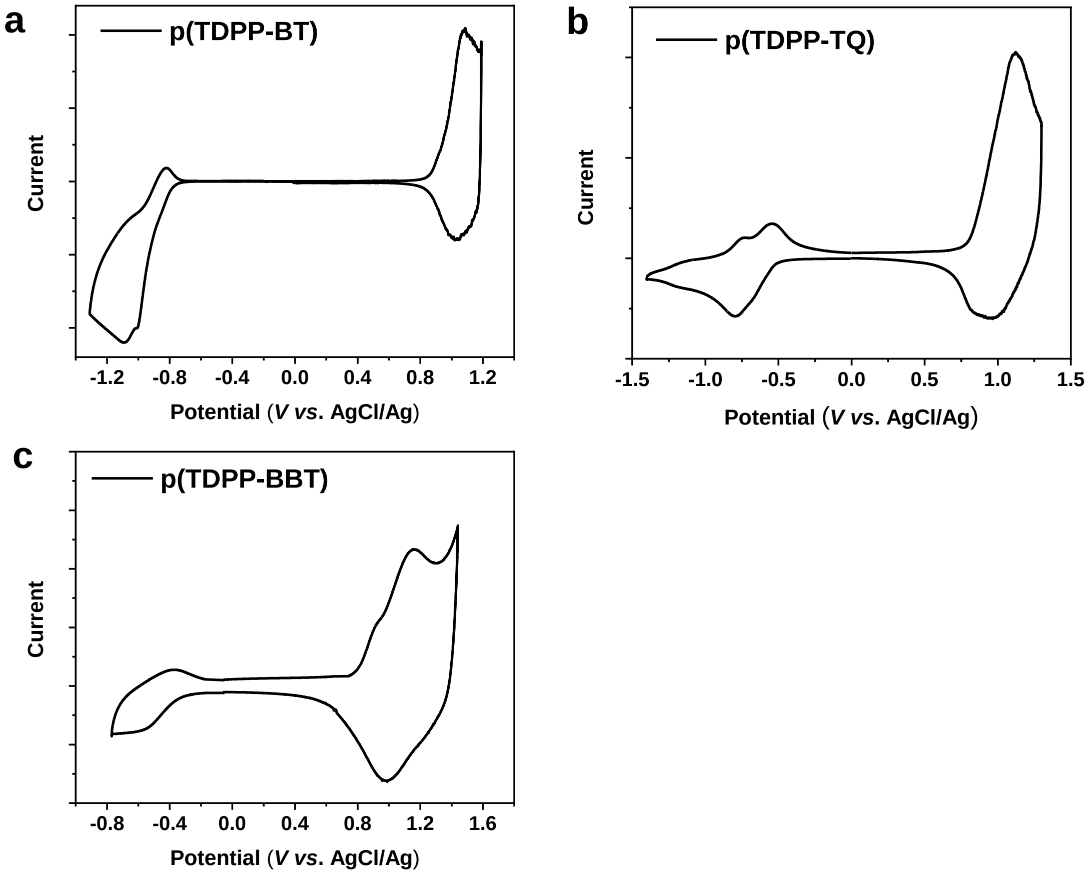


**Supplementary Figure 3.** Cyclic voltammograms of **a,** p(TDPP-BT), **b,** p(TDPP-TQ) and **c,** p(TDPP-BBT).

**Supplementary Table 2.** Inductively coupled plasma (ICP) emission spectroscopy of the polymers for trace metal analysis. No Fe was detected, and less than 0.1 wt% Pd and Sn were detected.

| | ICP results (ug/mL) | | | Metal concentration | | |
|---|---|---|---|---|---|---|
| Polymer | Fe | Pd | Sn | Fe | Pd | Sn |
| p(TDPP-BT)<br>(9.6 mg) | no | 0.27 | 0.32 | no | 0.003 mg,<br>0.03 wt% | 0.003 mg,<br>0.03 wt% |
| p(TDPP-TQ)<br>(10.0 mg) | no | 0.11 | 0.45 | no | 0.001 mg,<br>0.01 wt% | 0.005 mg,<br>0.05 wt% |
| p(TDPP-BBT)<br>(10.1 mg) | no | 0.30 | 0.68 | no | 0.003 mg,<br>0.03 wt% | 0.007 mg,<br>0.07 wt% |

## Supplementary Note 4. EPR and SQUID measurement

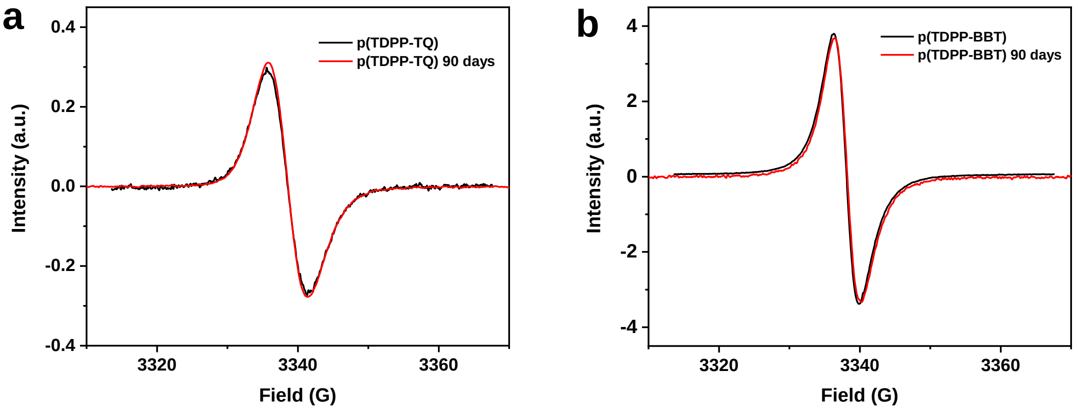


**Supplementary Figure 4. a,** Room temperature EPR of p(TDPP-TQ) and measured after 90 days. **b,** Room temperature EPR of p(TDPP-BBT) and measured after 90 days.

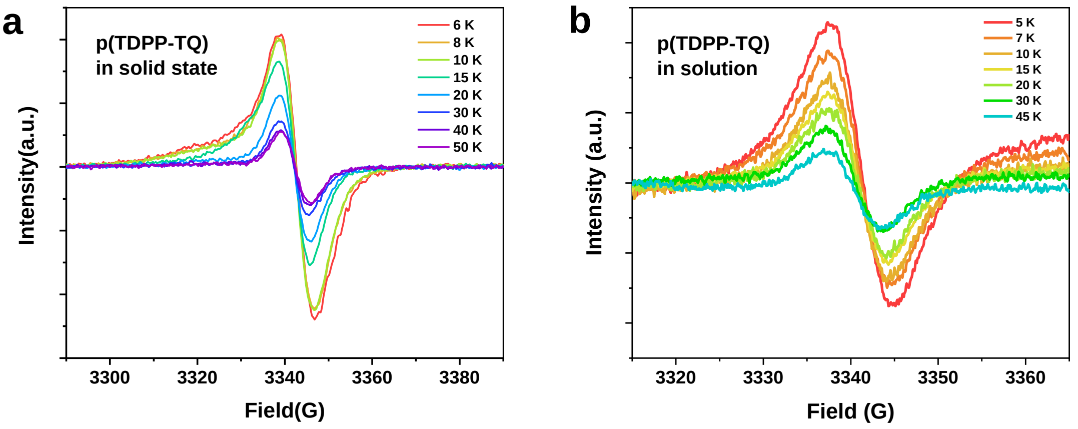


**Supplementary Figure 5.** Temperature dependent EPR intensity of **a,** p(TDPP-TQ) in solid state and **b,** in *o*-xylene solution.

## Quantitative EPR measurement for EPR spin susceptibility of p(TDPP-BBT)

Quantitative EPR measurement was performed and analysed to extract the EPR spin susceptibility[2]. Quantitative temperature-dependent EPR mearsurement of p(TDPP-BBT) were performed in 1.0 $10^{-3}$ M toluene:chloroform (3:1) solution (using a solution mixture to keep the solution in glass state) with a temperature rang from 130 K to 290 K (lower temperature will result in polymer pricipitation). A $1.0 \times 10^{-3}$ M

TEMPO solution in toluene:chloroform (3:1) was used as the standard reference for quantitative analysis. For each temperature point, EPR signal was recorded for p(TDPP-BBT) and TEMPO using the same instrument setting parameters. The double integrated intensity of the experimental spectrum was used to measure the number of spins. The relationship of the double integral of an experimental EPR spectrum and how it relates to the number of spins in the sample were calculated based on the following equation:

$$DI = c \cdot [G_R \cdot C_t \cdot n] \cdot [\frac{\sqrt{P} \cdot B_m \cdot Q \cdot n_B \cdot S \cdot (S+1)}{f(B_1, B_m)}] \cdot n_S \qquad \text{(Eq. 1)}$$

where $c$ is a constant input to the software from a sample with known number of spins, $G_R$ is receiver gain, $C_t$ is conversion time, $n$ is number of scans, $P$ is microwave power, $B_m$ is modulation amplitude, $Q$ is quality factor of resonator, $n_B$ is Boltzmann factor for temperature dependence, $S$ is total electron spin, $n_s$ is number of spins, $f(B_1, B_m)$ is spatial distribution of the microwave field and the modulation field experienced by the sample. For p(TDPP-BBT) and TEMPO, we control the sample properties and instrument setting parameters to keep $c$, $G_R$, $C_t$, $n$, $P$, $B_m$, $f(B_1, B_m)$ the same value or covert to the same value. We measured EPR spectrum of p(TDPP-BBT) and TEMPO at the same temperature, so $n_B$ is the same value. We asumed that p(TDPP-BBT) is a S = 1/2 system. The total number of spins for 30 μL 1.0 × 10$^{-3}$ M TEMPO solution $n_{s, \text{TEMPO}} = c^*V^*N_A = 3.0 \times 10^{-5} \times 1.0 \times 10^{-3} \times 6.0 \times 10^{23} = 1.8 \times 10^{16}$. So we can obtain the number of spins for p(TDPP-BBT) at each temperature point according the following equation:

$$n_{s,\,\text{p(TDPP-BBT)}} = \frac{DI_{\text{p(TDPP-BBT)}}}{DI_{\text{TEMPO}}} \cdot \frac{Q_{\text{TEMPO}}}{Q_{\text{p(TDPP-BBT)}}} \cdot n_{s,\,\text{TEMPO}} \qquad \text{(Eq. 2)}$$

The magnetic susceptibility can be calculated by the following equation:

$$\chi = \mu_0 \frac{\hbar^2 \gamma_e^2}{3k_B T} S(S+1) \cdot n_{s,\,\text{p(TDPP-BBT)}} \qquad \text{(Eq. 3)}$$

where $\mu_0 = 4\pi \times 10^{-7}$ T$^2$ J m$^3$, $\gamma_e = 1.7608 \times 10^{11}$ s$^{-1}$ T$^{-1}$, $\hbar = 1.0546 \times 10^{34}$ J s, $S = 1/2$, $n_{s,\,\text{p(TDPP-BBT)}}$ is the number of spins, $k_B = 1.3806 \times 10^{-23}$ J K$^{-1}$. The relationship of the product of magnetic susceptibility and temperature ($\chi T$) to temperature ($T$) is shown in

Supplementary Fig. 6. The data were fitted linearly with the equation $\chi_{\text{total}}T = C + \chi_{\text{Pauli}}T$, where $C$ is Curie constant. The fitted result is $\chi_{\text{Pauli}} = 3.9 \pm 0.9 \times 10^{-4}$ cm$^3$ mol$^{-1}$, $C$ = 0.13 ± 0.019 cm$^3$ mol$^{-1}$ K.

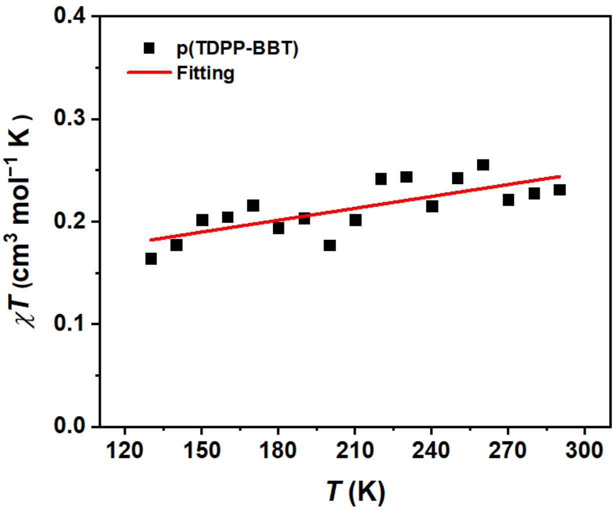


**Supplementary Figure 6.** $\chi T$ versus temperature ($T$) plot of p(TDPP-BBT) obtained from quantitative temperature-dependent EPR measurement, which is used for $\chi_{\text{Pauli}}$ and $C$ calculations.

In the manuscript, we obtained Pauli paramagnetic susceptibility, $\chi_{\text{Pauli}} = 3.5 \times 10^{-3}$ cm$^3$ mol$^{-1}$ from SQUID measurement, while the Curie constant is $C = 4.0 \times 10^{-2}$ cm$^3$ mol$^{-1}$ K.

The two $\chi T$-$T$ relationships of p(TDPP-BBT) obtained from SQUID and EPR are different because the state of the p(TDPP-BBT) sample is different. For SQUID measurement we used solid-state samples, while quantitative EPR was performed in diluted solution. Note that quantitative EPR cannot be performed in the solid state because of the strong spin-spin interactions. The following Supplementary Fig. 7 shows the double integral (DI) of the solid-state EPR signals of the reference compound TEMPO, which clearly deviate from the expected $S$ = 1/2 behavior that DI*T (red solid squares) should be a constant value.

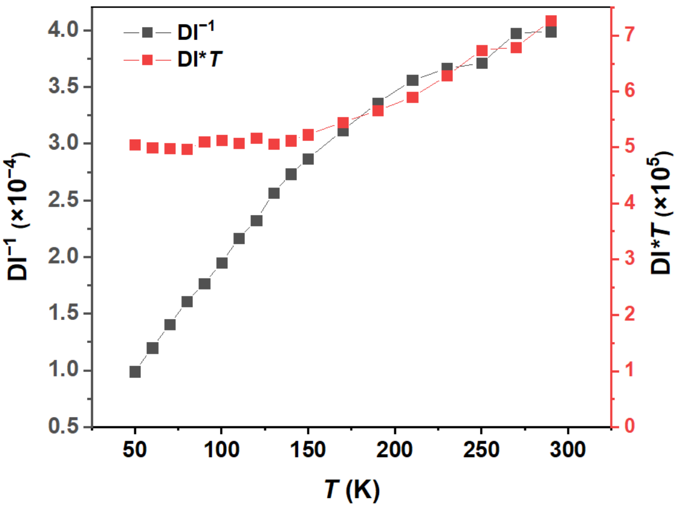


**Supplementary Figure 7.** The temperature-dependent double integral$^{-1}$ (DI$^{-1}$) and DI*$T$ of the solid-state EPR of the reference compound TEMPO.

In the solid state, the Pauli paramagnetic component is larger than Curie paramagnetic component of $\chi T$ even at low temperature (suppose at 20 K, $\chi_{\mathrm{Pauli}}T = 7.0 \times 10^{-2}$ cm$^3$ mol$^{-1}$ is large than C = $4.0 \times 10^{-2}$ cm$^3$ mol$^{-1}$ K), which is consistent with the strong interchain interactions and good charge delocalization in p(TDPP-BBT). However, the Curie paramagnetic component is comparable to the Pauli paramagnetic component $\chi T$ at room temperature in diluted solution (suppose at 300 K, $\chi_{\mathrm{Pauli}}T = 0.12 \pm 0.027$ cm$^3$ mol$^{-1}$ K while $C = 0.13 \pm 0.019$ cm$^3$ mol$^{-1}$ K). We would like to note that the quantitative temperature-dependent EPR measurement will also underestimate the Pauli paramagnetic component because the $\chi$ is calculated based on the premise that the ground state has a large separation in energy from the first excited states[3] while our p(TDPP-BBT) molecule has a very small energy separation between singlet and triplet state. Therefore, the SQUID measurement and the quantitative EPR measurement are consistent. SQUID provides a description of the solid-state behavior of p(TDPP-BBT), while EPR reflects the less aggregated solution behavior.

We attributed the origin of the Pauli susceptibility in our polymers to the small bandgap of these polymers. The small bandgap provides the polymers with free charge carriers in the solid state. Both p(TDPP-TQ) and p(TDPP-BBT) are conductive at zero gate voltage. We summarized the magnitude of the Pauli susceptibility and the

calculated $N(E_F)$ for doped conjugated polymers, neutral radicals and our polymer in Supplementary Table 3. For doped polymers, the Pauli susceptibility and the calculated $N(E_F)$ are in the range of $10^{-5}$–$10^{-4}$ emu mol$^{-1}$ and $10^{20}$–$10^{21}$ eV$^{-1}$ cm$^{-3}$, respectively[4, 5, 6]. Interestingly, in organic small-molecule radicals, the values of Pauli susceptibility and $N(E_F)$ are clearly larger than doped polymers. The $N(E_F)$ values of are in the range of $10^{22}$–$10^{23}$ eV$^{-1}$ cm$^{-3}$, though the molecules are not highly conductive[7, 8, 9]. For our polymers, the Pauli susceptibility and the $N(E_F)$ are similar to those organic small-molecule radicals. Haddon *et al.* attributed the larger Pauli susceptibility in the organic radicals to the strong electron-electron interactions[7, 10]. However, further investigations are needed to better understand the larger Pauli susceptibility in organic radicals and our high-spin ground-state polymers, but are beyond the scope of this work.

**Supplementary Table 3.** Comparison of the Pauli susceptibility ($\chi_{Pauli}$) and DOS at the Fermi level ($N(E_F)$) of the doped conjugated polymers (Entry 1-3), small molecule radicals (Entry 4-6), and our polymers (Entry 7-8).

| Entry | Compounds | $\chi_{Pauli}$ (emu mol$^{-1}$) | $N(E_F)$ (eV$^{-1}$ cm$^{-3}$) | Reference |
|---|---|---|---|---|
| 1 | $AsF_5^-$ doped polyacetylene | $3.2 \times 10^{-5}$ | $4.6 \times 10^{21}$ | [4] |
| 2 | FTS doped PBTTT | $3.9 \times 10^{-5}$ | $9.4 \times 10^{20}$ | [5] |
| 3 | $F_4$-TCNQ doped PBTTT | $2.4 \pm 0.2 \times 10^{-4}$ | $5.7 \pm 0.5 \times 10^{21}$ | [6] |
| 4 | Phenalenyl-based neutral radical | $5.0 \times 10^{-4}$ | $1.6 \times 10^{22}$ | [7] |
| 5 | Phenalenyl-based neutral radical | $4.5 \times 10^{-4}$ | $1.8 \times 10^{22}$ | [8] |
| 6 | Ladder-type pernigraniline salt | $1.2 \times 10^{-2}$ | $1.9 \times 10^{23}$ | [9] |
| 7 | p(TDPP-TQ) | $8.2 \times 10^{-4}$ | $1.5 \times 10^{22}$ | This work |
| 8 | p(TDPP-BBT) | $3.5 \times 10^{-3}$ | $6.5 \times 10^{22}$ | This work |

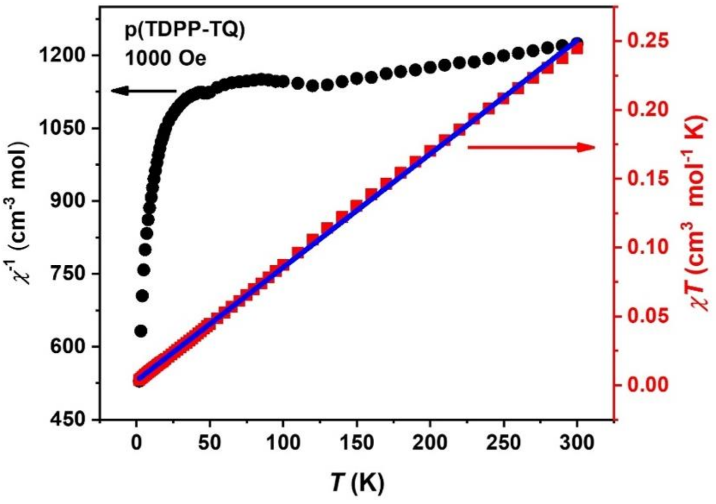


**Supplementary Figure 8.** Variable temperature magnetic susceptibility of p(TDPP-TQ) from 2 K to 300 K. Solid squares are the experimental data, solid line is the fitting line from 50 K to 300 K.

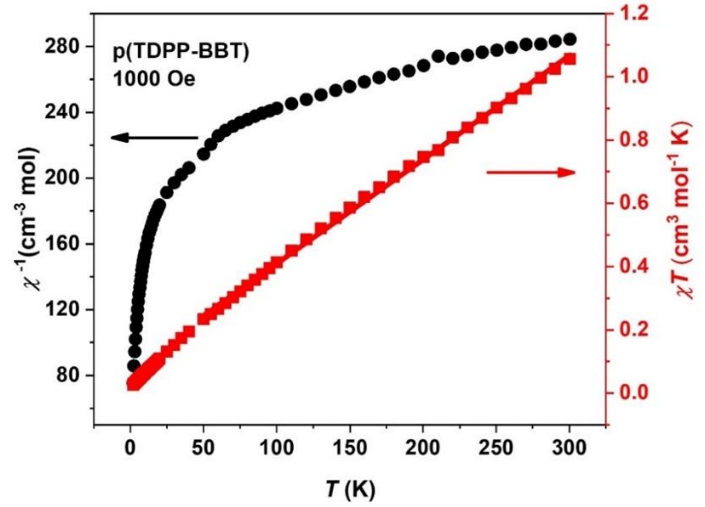


**Supplementary Figure 9.** Variable temperature magnetic susceptibility of p(TDPP-BBT) from 2 K to 300 K. Solid squares are the experimental data, solid line is the fitting line from 50 K to 300 K.

## Supplementary Note 5. Temperature-dependent UV-vis absorption spectra of p(TDPP-TQ) and p(TDPP-BBT)

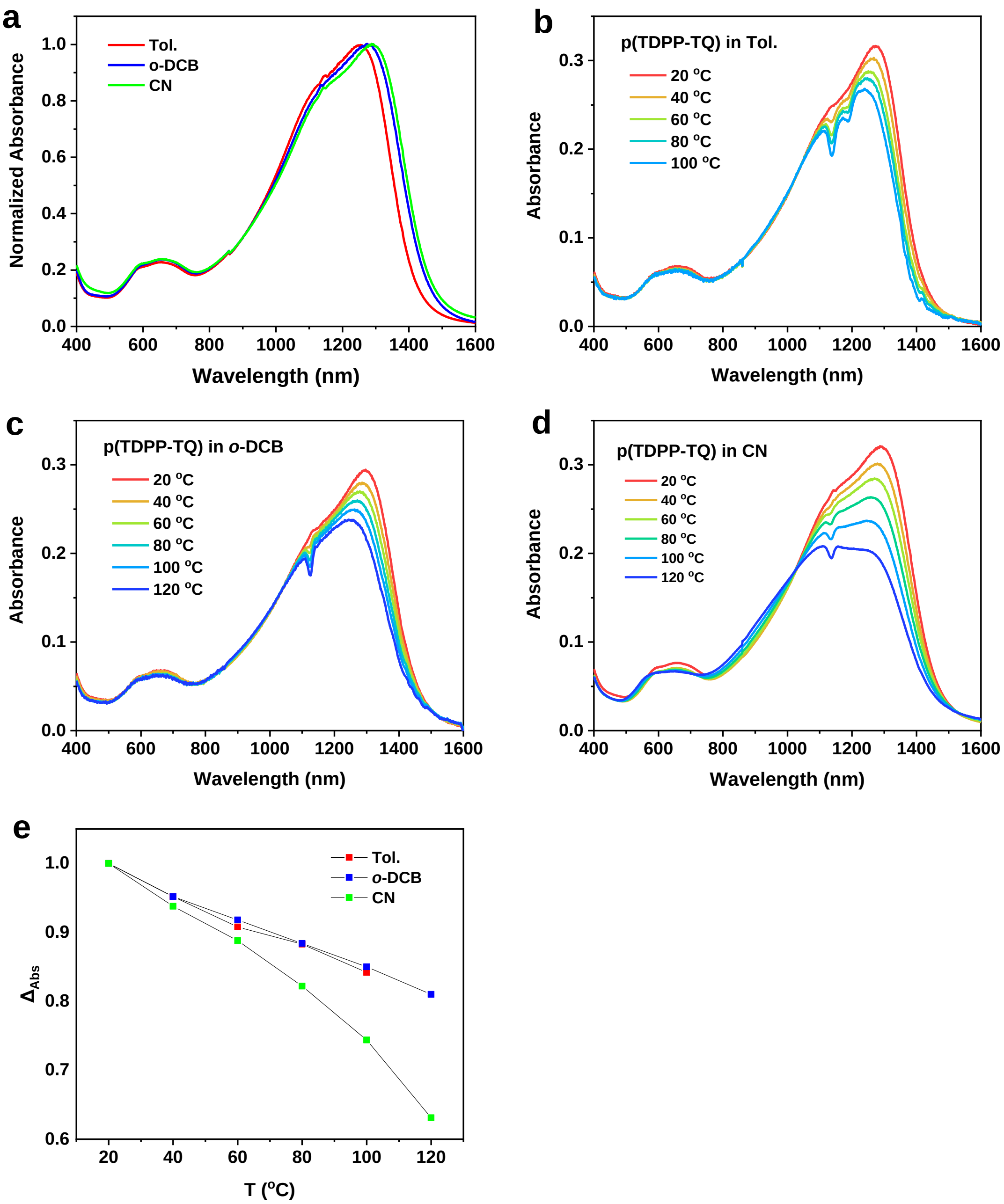


**Supplementary Figure 10. UV-vis-NIR absorption spectra of p(TDPP-TQ) at a concentration of 1×10$^{-5}$ M. a,** in different solvent of toluene (Tol.), *o*-dichlorobenzene (*o*-DCB), 1-chloronaphthalene (CN), **b,** in toluene at different temperature, **c,** in *o*-dichlorobenzene at different temperature, **d,** in 1-CN at different temperature. **e,** p(TDPP-TQ) shows similar absorption behavior but different $\lambda_{max}$ which indicate different aggregation properties among the solvents. The temperature dependent absorption between different solvents show the tendency of the disaggregation as increasing the temperature. In 1-CN, p(TDPP-TQ) showed much clear disaggregation phenomenon compared to its toluene and *o*-dichlorobenzene.

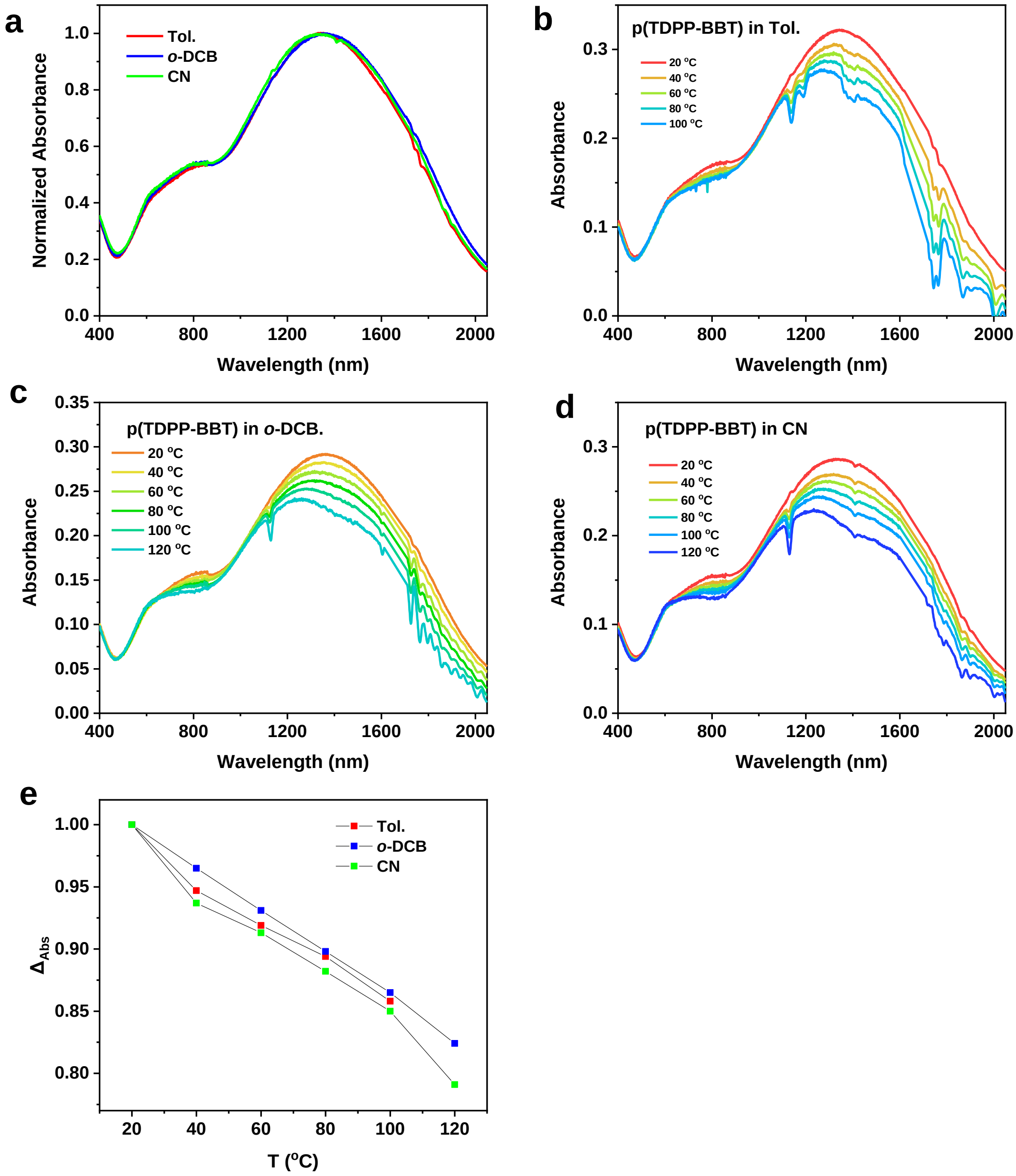


**Supplementary Figure 11. UV-vis-NIR absorption spectra of p(TDPP-BBT) at a concentration of 1 × 10$^{-5}$ M. a,** in different solvent of chlorobenzene (CB), *o*-dichlorobenzene (*o*-DCB), 1-chloronaphthalene (CN), **b,** in chlorobenzene at different temperature, **c,** in *o*-dichlorobenzene at different temperature, **d,** in 1-chloronaphthalene at different temperature. **e,** p(TDPP-BBT) shows similar absorption behavior and no clear $\lambda_{max}$ difference among different solvent. The temperature dependent absorption between different solvents show the similar tendency of aggregation.

## Supplementary Note 6. DFT calculations of oligomers and dimers

Theoretical calculation was performed in Gaussian 16 to investigate the relationship of structure and properties. The molecule structure optimization of close-

shell and open-shell singlet and triplet was conducted in the gas phase using 6-31G** basis set. For close-shell singlet, B3LYP was used. For open-shell, the long-range corrected spin-unrestricted density functional theory, LC-UBLYP and broken-symmery approach were used. The repeat number of monomers is from 1 to 6. The alkyl chains were truncated to methyl group for simpilicity. The $\Delta E_{S\text{-}T}$ was calculated from the energy difference between open-shell singlet and triplet. Diradical character index ($y_0$) was calculated by the following equations:

$$y_0 = 1 - \frac{2T}{1+T^2}, \; T = \frac{n_{\mathrm{HOMO}} - n_{\mathrm{LUMO}}}{2} \qquad \text{(Eq. 4)}$$

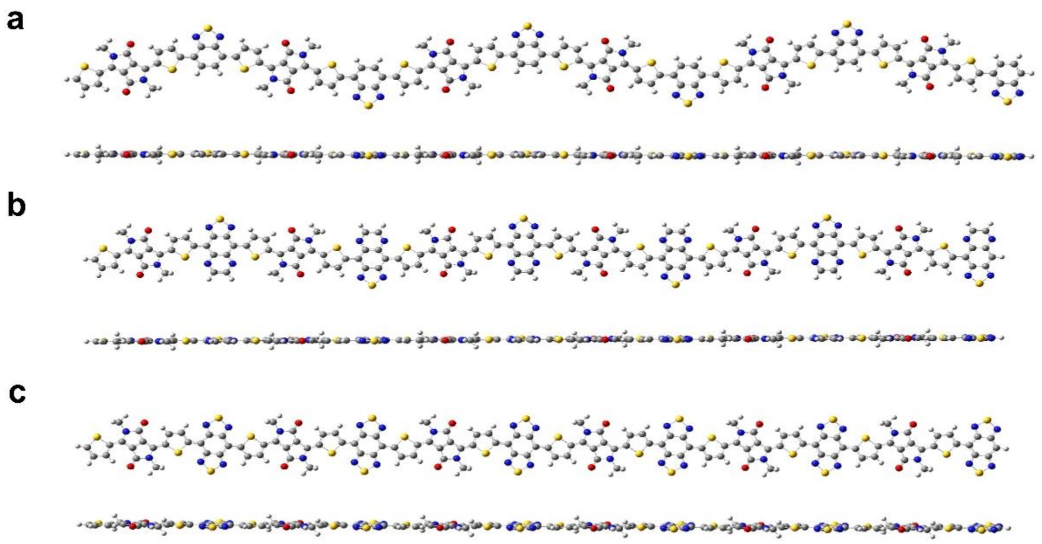

**Supplementary Figure 12.** Top view and side view of DFT-optimized close-shell geometries of **a,** (TDPP-BT)$_6$, **b,** (TDPP-TQ)$_6$, **c,** (TDPP-TQ)$_6$. All three polymers show very good planarity.

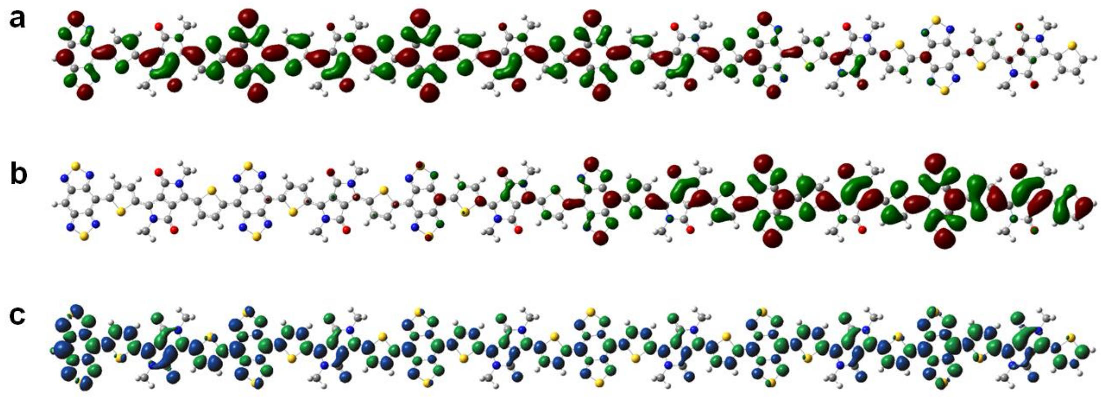


**Supplementary Figure 13.** DFT calculated molecular frontier orbitals and spin density distribution of $(TDPP-BBT)_6$. $(TDPP-BBT)_6$ also exhibits similar good planarity. **a,** α-SOMO and **b,** β-SOMO **c,** spin density distribution of the open-shell singlet. The localization of the α and β singly occupied MOs (SOMOs) of the singlet is on the opposite sides of the oligomer.

**Supplementary Table 4.** Calculated $E_{\mathrm{LUMO}}$-$E_{\mathrm{HOMO}}$, $\Delta E_{\mathrm{S\text{-}T}}$, $y_0$ values for $(TDPP-BT)_n$ oligomers.

| n | $E_{\mathrm{LUMO}}$-$E_{\mathrm{HOMO}}$ | $\Delta E_{\mathrm{S\text{-}T}}$ | $y_0$ |
|---|---|---|---|
| 1 | 47.57 | −20.50 | 0 |
| 2 | 36.44 | −16.71 | 0.0031 |
| 3 | 32.97 | −15.48 | 0.0077 |
| 4 | 31.44 | −15.33 | 0.011 |
| 5 | 30.86 | −15.26 | 0.012 |
| 6 | 30.70 | −15.22 | 0.012 |

**Supplementary Table 5.** Calculated $E_{LUMO}$-$E_{HOMO}$, $\Delta E_{S\text{-}T}$, $y_0$ values for (TDPP-TQ)$_n$ oligomers.

| n | $E_{LUMO}$-$E_{HOMO}$ | $\Delta E_{S\text{-}T}$ | $y_0$ |
|---|---|---|---|
| 1 | 34.15 | −13.56 | 0.0088 |
| 2 | 23.89 | −5.26 | 0.16 |
| 3 | 17.50 | −2.49 | 0.23 |
| 4 | 18.78 | −0.70 | 0.29 |
| 5 | 14.64 | −0.61 | 0.33 |
| 6 | 14.12 | −0.54 | 0.35 |

**Supplementary Table 6.** Calculated $E_{LUMO}$-$E_{HOMO}$, $\Delta E_{S\text{-}T}$, $y_0$ values for (TDPP-BBT)$_n$ oligomers.

| n | $E_{LUMO}$-$E_{HOMO}$ | $\Delta E_{S\text{-}T}$ | $y_0$ |
|---|---|---|---|
| 1 | 28.38 | −7.60 | 0.10 |
| 2 | 15.71 | −2.30 | 0.39 |
| 3 | 10.12 | −0.80 | 0.61 |
| 4 | 3.73 | −0.41 | 0.79 |
| 5 | 6.49 | −0.24 | 0.98 |
| 6 | 5.65 | −0.16 | 0.99 |

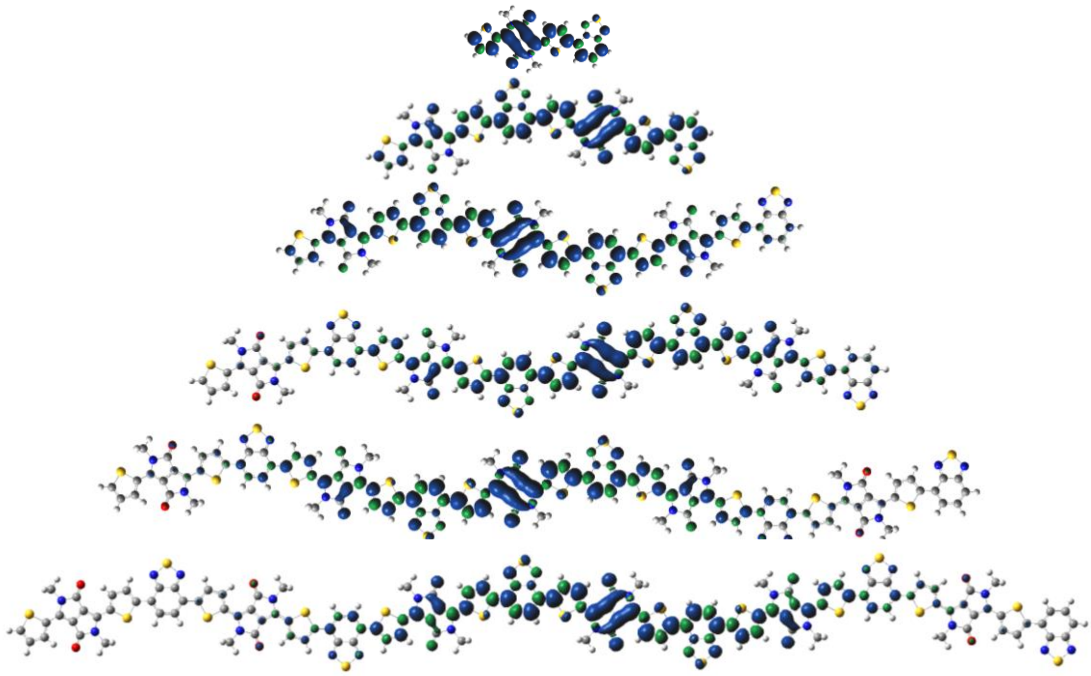

**Supplementary Figure 14**. Spin density distribution of the triplet states of the (TDPP-BT)$_n$ (n = 1-6) oligomers. The spin tends to distribute in the middle of the oligomers.

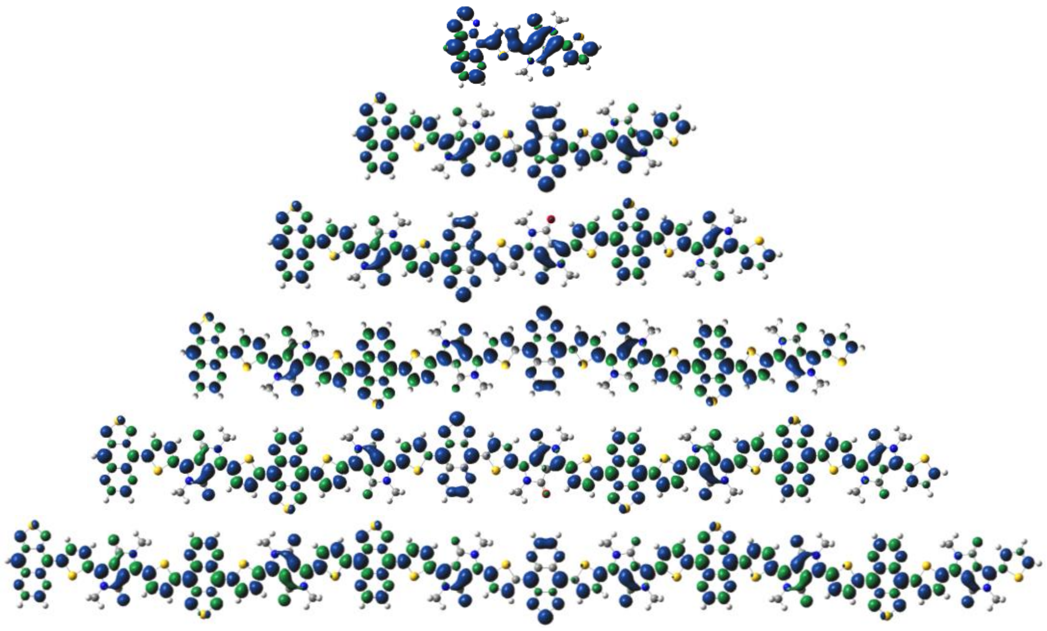

**Supplementary Figure 15.** Spin density distribution of the triplet states of the (TDPP-TQ)$_n$ oligomers (n = 1-6). As the number n increases, the spin tends to distribute along the whole backbone. The spin density distribution in (TDPP-TQ)$_n$ (n = 1-6) clearly indicates the high degree of delocalization of the spin.

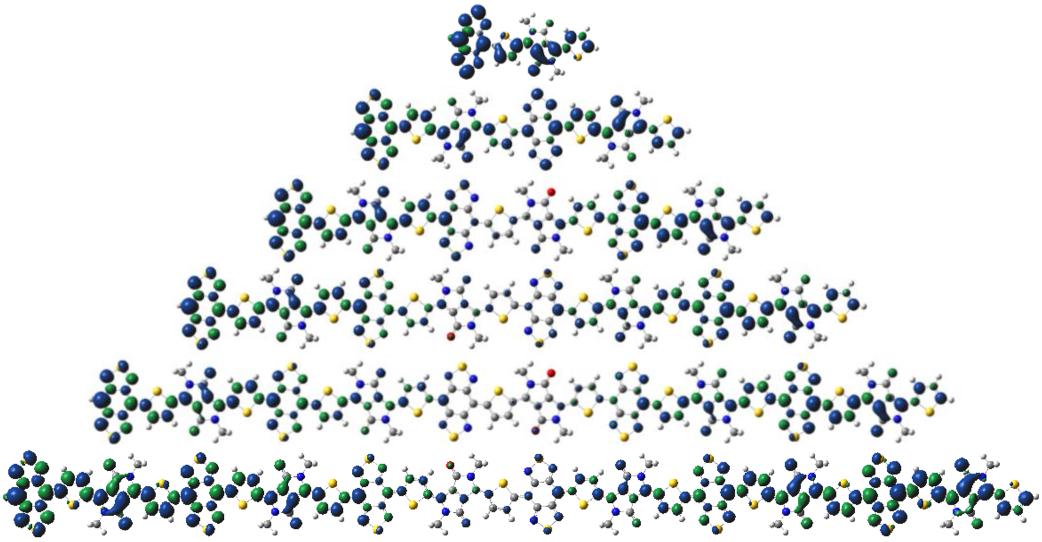

**Supplementary Figure 16.** Spin density distribution of the triplet states of the (TDPP-BBT)$_n$ oligomers (n = 1-6). As the number n increases, the spin tends to distribute on the both ends of the oligomers.

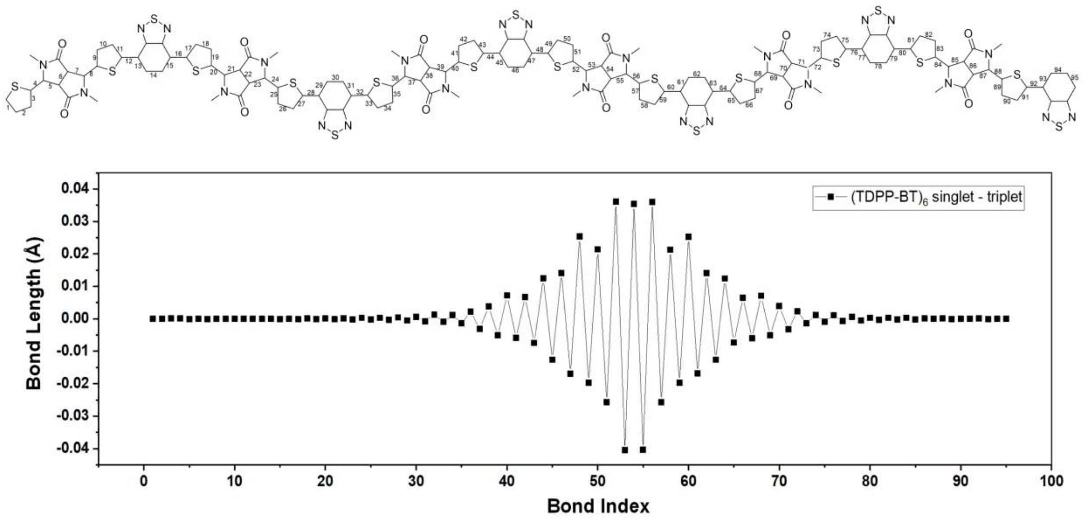


**Supplementary Figure 17.** Bond length alternation (BLA) analysis of (TDPP-BT)$_6$. The chart in the bottom is the bond length difference between open-shell singlet and triplet. The result shows that only in the middle of the oligomer, the bond lengths of open-shell singlet state and triplet state are different, which is consistent with the spin density distribution trendency of the triplet state.

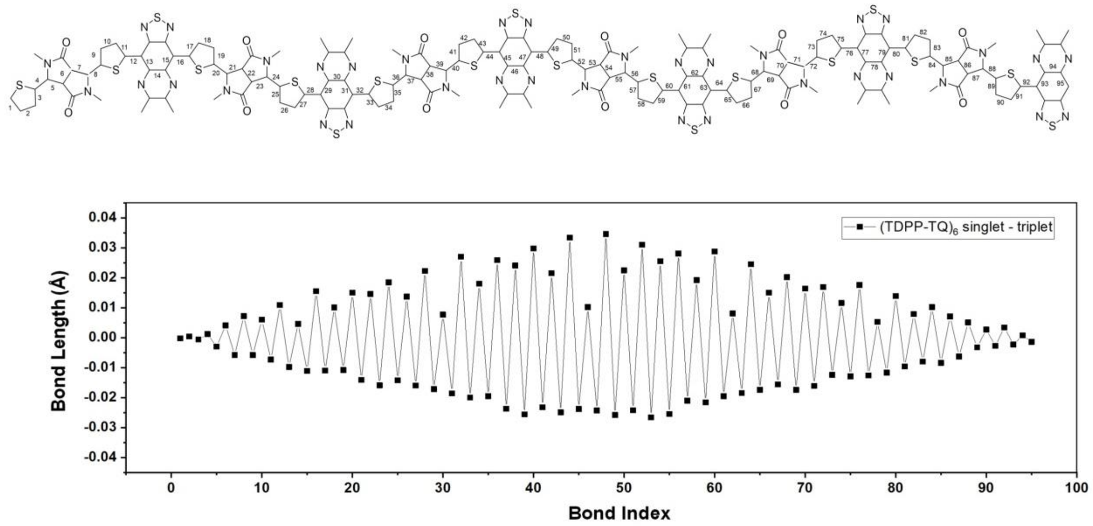


**Supplementary Figure 18.** BLA analysis of $(TDPP\text{-}TQ)_6$. The chart in the bottom is the bond length difference between open shell singlet and triplet. In the entire oligomer molecule, the bond lengths of open-shell singlet state and triplet state are different, which is consistent with the spin density distribution trendency of the triplet that the spin delocalized in the entire molecule.

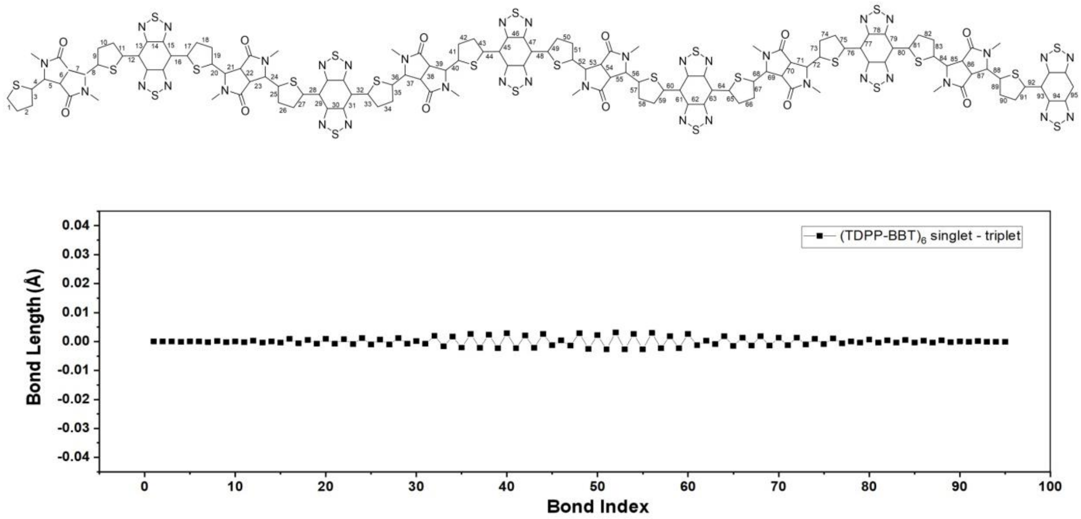


**Supplementary Figure 19.** BLA analysis of $(TDPP\text{-}BBT)_6$. The chart in the bottom is the bond length difference between open shell singlet and triplet. The bond length difference between the open-shell state and the singlet state is very small, indicating that the structure difference between the singlet state and the triplet state is very small. This corresponds to the small difference in energy levels between the singlet state and the triplet state.

To estimate the intermolecular interaction, we performed DFT calculations to compare the energy, spin density, and molecular orbitals between a single polymer chain and dimers with different interchain packings. Since the computational costs of a large number of atoms are pretty high, the following discussions are based on two π-stacked polymer chains made of oligomers with 4 repeat units. As shown in Supplementary Fig. 20, polymer chains are arranged in a cofacial packing with different degrees of translation of one polymer chain along the long axis (x-axis, translated 1 Å for each step). We have tried to perform structure optimization of the two stacked chains initially. However, the polymer chains were bent significantly and deviated from the real molecular packing from X-ray data in the solid state because only dimers are included here and we omit the complex multiple and multi-directional intermolecular interactions in solid state. Thus, we calculate the single-point energies of each cofacial dimer without structure optimization.

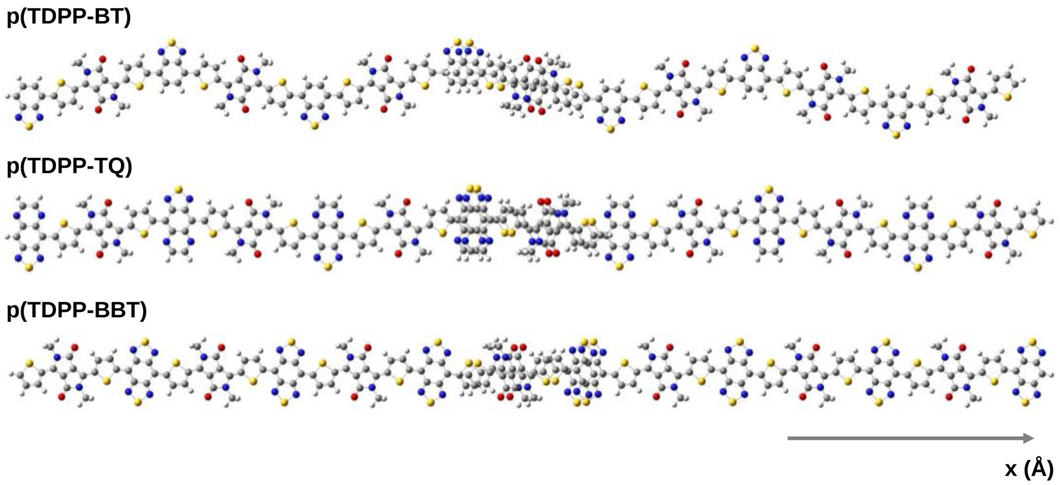


**Supplementary Figure 20.** Representation of the co-facially stacked polymer chains with different degrees of translation of one polymer chain along the long axis (x-axis). The above alignments were defined as the original position. The single polymer chain on the top was translated along the x-axis by 1Å each step at a fixed π-π stacking distance of 3.5 Å to search for the optimal packing conformation.

As expected, all the stacked dimers are more stable than two single chains, indicating the strong π-π stacking interactions between the polymer chains (Supplementary Fig. 21a). Both p(TDPP-TQ) and p(TDPP-BBT) showed larger

binding energy than p(TDPP-BT), which could be ascribed to the larger aromatic rings of TQ and BBT moieties and their linear backbones. We also calculated the singlet-triplet energy gap $\Delta E_{\text{S-T}}$ of the dimers with different degrees of translation. The maximum $\Delta E_{\text{S-T}}$ of the dimers are −14.1 kcal $mol^{-1}$ for p(TDPP-BT), −2.87 kcal $mol^{-1}$ for p(TDPP-TQ), and 4.95 kcal $mol^{-1}$ for p(TDPP-BBT), which show a similar trend as those of the single chains (as mentioned in the manuscript). These results indicate that the p(TDPP-BBT) dimers could have a more stable triplet ground state than the single chain. Interestingly, the $\Delta E_{\text{S-T}}$ value does not change significantly except for certain packing conformations (Δx = 0 Å for p(TDPP-TQ); Δx = 2 Å for p(TDPP-BBT); Δx = 4 Å for p(TDPP-BT)).

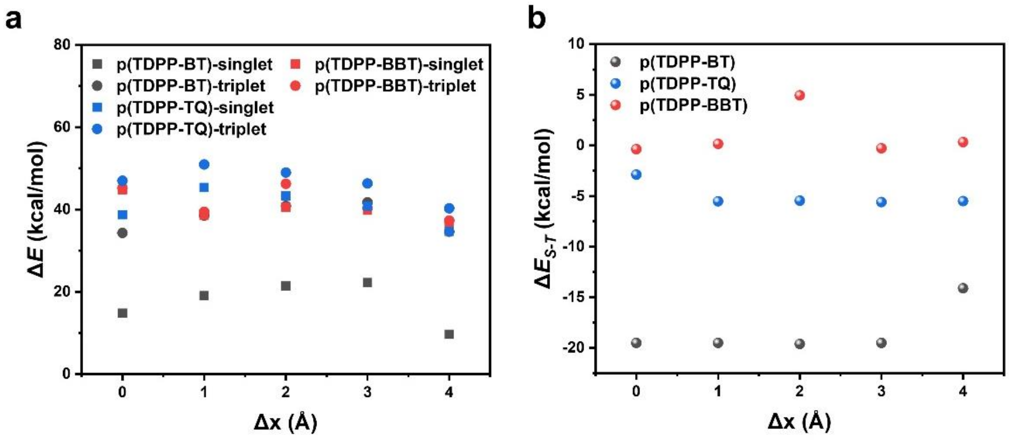


**Supplementary Figure 21. a**, Evolution of the binding energy of the cofacially stacked polymer chains with different degrees of translation of one polymer chain along the long axis. The binding energy is calculated based on $\Delta E_{\text{singlet}} = 2\times E_{\text{single-chain-singlet}} - E_{\text{dimer-singlet}}$(or $\Delta E_{\text{triplet}} = 2\times E_{\text{single-chain-triplet}} - E_{\text{dimer-triplet}}$). **b**, Evolution of the $\Delta E_{\text{S-T}}$ value of the cofacially stacked polymer chains with different degrees of translation. All DFT calculations were performed at the UB3LYP/6-31G(d) level with Grimme's D3BJ dispersion correction.

p(TDPP-BT) dimers showed a large negative $\Delta E_{\text{S-T}}$ value, making it hard to access the triplet state. This is consistent with our result that p(TDPP-BT) is a close-shell polymer with a singlet ground state. We then focus on the difference between p(TDPP-BBT) and p(TDPP-TQ). As shown in Supplementary Fig. 22, the frontier orbitals of p(TDPP-BBT) and p(TDPP-TQ) dimers have shown obvious overlaps between two chains, consistent with their strong interchain interactions shown in Supplementary Fig.

21a. The large spatial overlap between frontier orbitals is responsible for the admixture of a doubly excited configuration into the ground state, that is, a singlet biradical character[11].

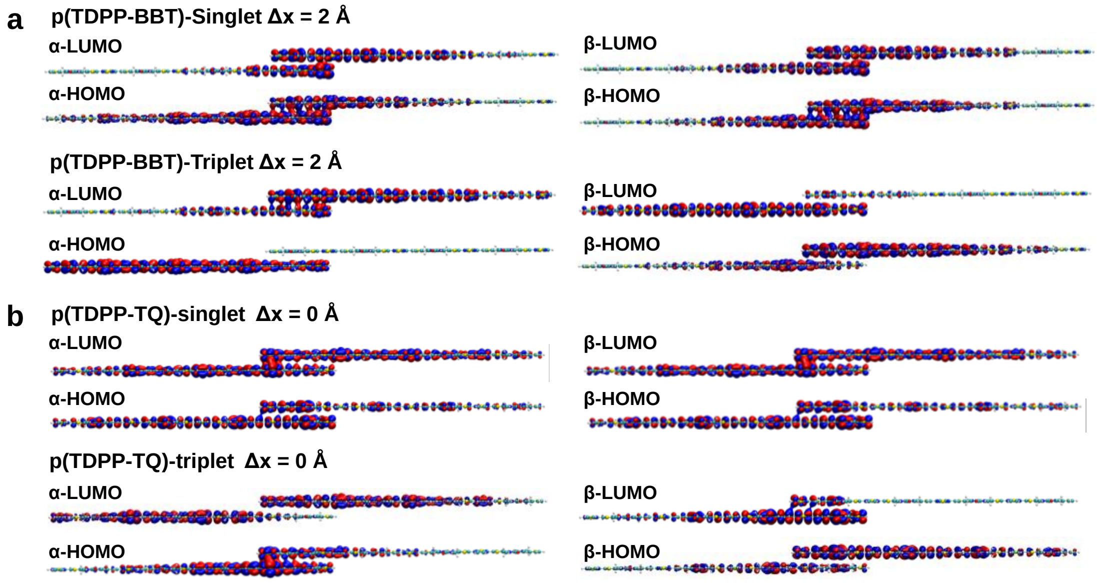


**Supplementary Figure 22.** Isosurfaces (0.008 a.u.) of the frontier orbitals of p(TDPP-BBT) and p(TDPP-TQ) dimers at their singlet and triplet state.

The spin density of the p(TDPP-BBT) single chain distributes at both ends of the oligomer (Supplementary Fig. 23a). However, the spin density of the overlap region in the p(TDPP-BBT) dimer decreased or even disappeared and the spin density mainly distributed at the two ends of the dimer for both singlet and triplet states (red circle in Supplementary Fig. 23a). These results agree well with our assumption that intermolecular antiferromagnetic spin-spin interactions might exist through interchain π-π stacking.

The spin density of p(TDPP-TQ) at singlet state is very small that can not be visualized at 0.003 a.u. isosurface. The spin density distributions for its triplet state are shown in Supplmentary Fig. 23b. Spin density redistribution is also observed for p(TDPP-TQ), indicating the existence of strong intermolecular spin-spin interaction. Interestingly, the two polymers exhibit significantly different spin distributions. The intermolecular electron coupling through π-π stacking decreases the spin density in the

middle of the p(TDPP-BBT) dimer. In other words, this feature increases the distance of the two unpaired spins that located at the end of the dimer and diminishes the electron exchange interaction, leading to the formation of doublets. However, for p(TDPP-TQ), the spin is distributed all along the polymer chain for both single chain and dimer. The blue circles in Supplementary Fig. 23b indicate that the spin-up in the single-chain turns into mixed up- and down-spin states in the dimer, suggesting the strong electron exchange interaction between the spins. Therefore, with the antiferromagnetic spin-spin interactions, the two spins are far away from each other in p(TDPP-BBT) dimer, becoming two independent radicals (or biradicals) and exhibiting a doublet ground state. While for p(TDPP-TQ), the two radicals are closer with stronger interactions, forming diradicals and exhibiting triplet ground state in solid-state.

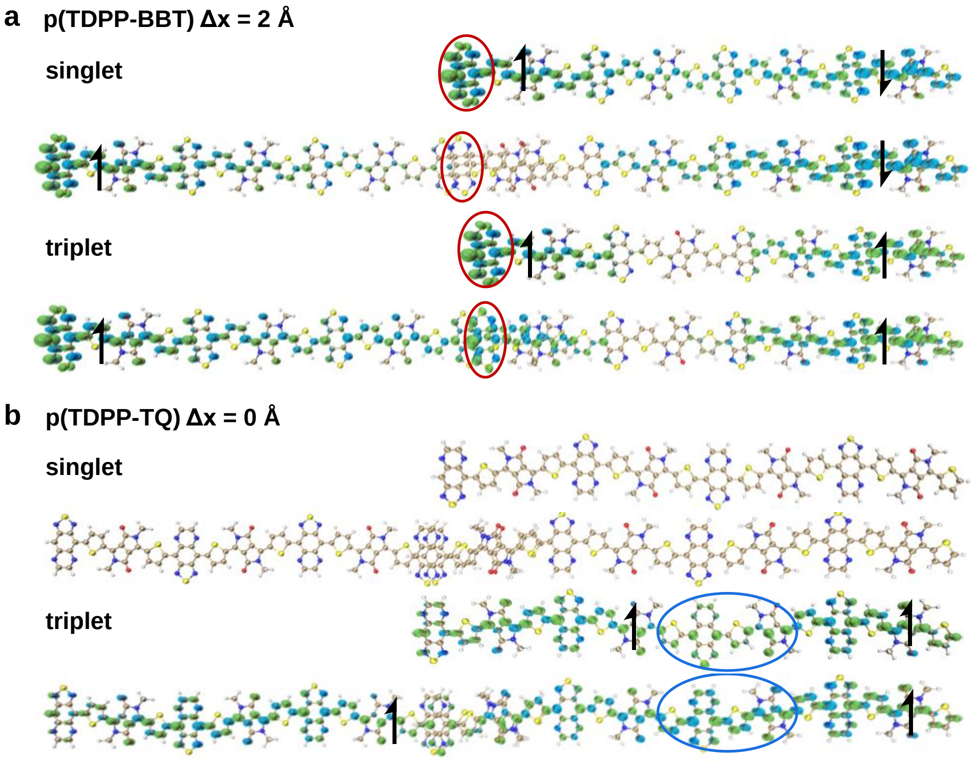


**Supplementary Figure 23.** Spin distributions of the single-chain and cofacial dimers of **a**, p(TDPP-BBT) and **b**, p(TDPP-TQ) at their singlet and triplet states. The isosurface value is 0.003 a.u. (UB3LYP/6-31G(d) with Grimme's D3BJ dispersion correction). The isosurface in lime and cyan denotes positive and negative spin density, corresponding to the up- and down-spin states, respectively.

**Supplementary Note 7. Device Fabrication and Characterization**

**Field effect transistor (FET) devices fabrication and characterization.**

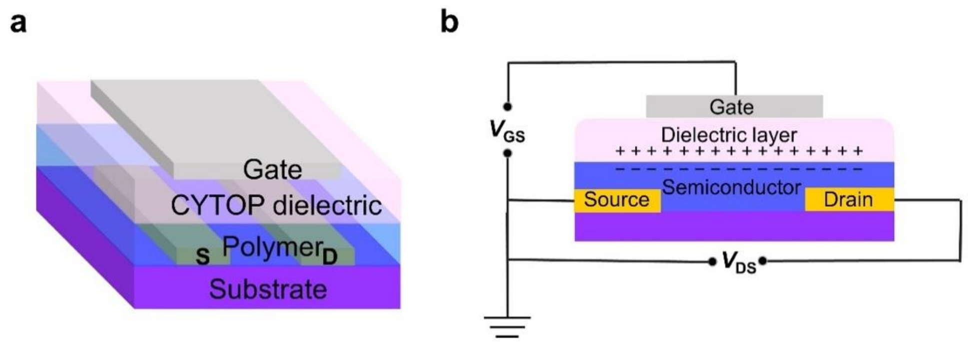


**Supplementary Figure 24. a,** Schematic diagram and **b,** circuit diagram of top-gate/bottom-contact OFET device structure.

Top-gate/bottom-contact FET devices were fabricated to characterize the charge carrier mobility of the polymers. The source and drain electrodes (Ti 2 nm/Au 40 nm) were patterned on $SiO_2$ substrate by photolithography and lift-off process. The substrates were cleaned by using ultrasonication in acetone, cleaning agent, deionized water (three times), and isopropanol. Thin polymer film was deposited by spin-coating at 1500 rpm for 60 s by using the polymer solution of 3 g/L in 1-chloronaphthalene (CN). The polymer film was first baked at 100 °C and then annealed at 150 °C. After depositing the polymer film, a CYTOP solution (CTL809M/CT-solv 180 =3/1) was spin-coated on top of the polymer film at 2000 rpm for 60 s and annealed at 100 °C, yielding a dielectric layer of 500 nm thick. An aluminum layer of 50 nm was evaporated onto the dielectric layer as the gate electrode. The FET devices were measured on a probe stage under ambient conditions using Keithley 4200 SCS Parameter Analyzer.

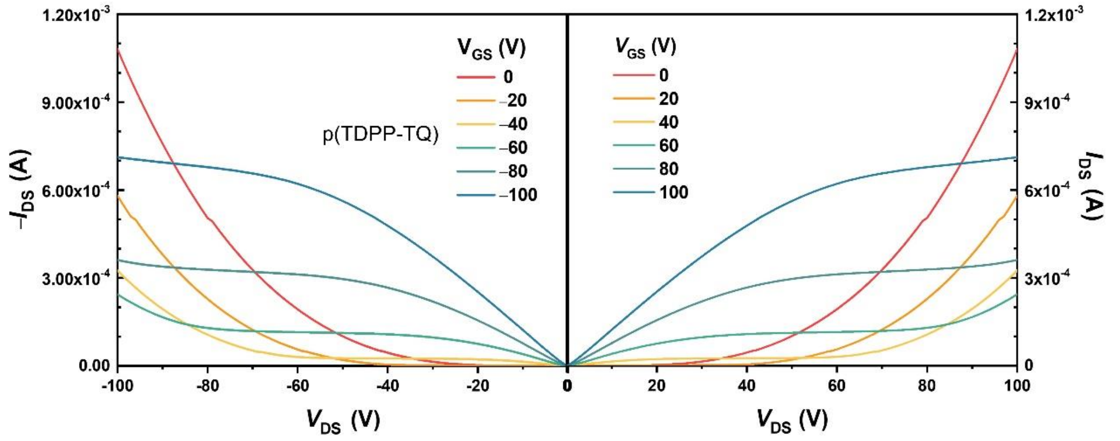


**Supplementary Figure 25.** Typical output characteristics of p(TDPP-TQ) OFET devices were fabricated in glovebox and tested in ambient conditions ($L$ = 10 μm, $W$ = 200 μm). 500 nm thick CYTOP was used as the dielectric layer ($C_i$ = 3.7 nF cm$^{-2}$).

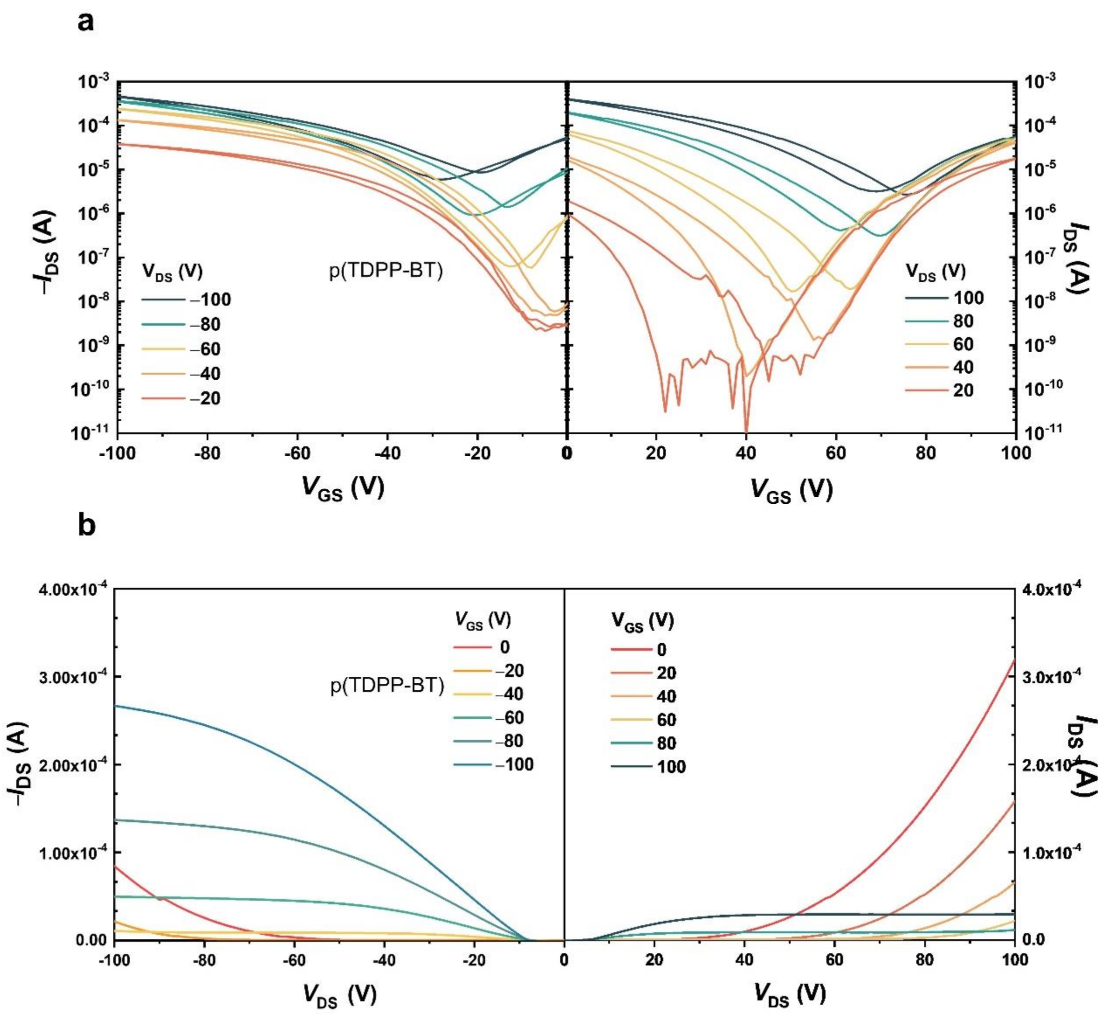


**Supplementary Figure 26. a,** Typical transfer and **b,** output characteristics of a typical p(TDPP-BT) OFET devices fabricated in glovebox and tested in ambient conditions ($L$ = 10 μm, $W$ = 200 μm). 500 nm thick CYTOP was used as the dielectric layer ($C_i$ = 3.7 nF cm$^{-2}$).

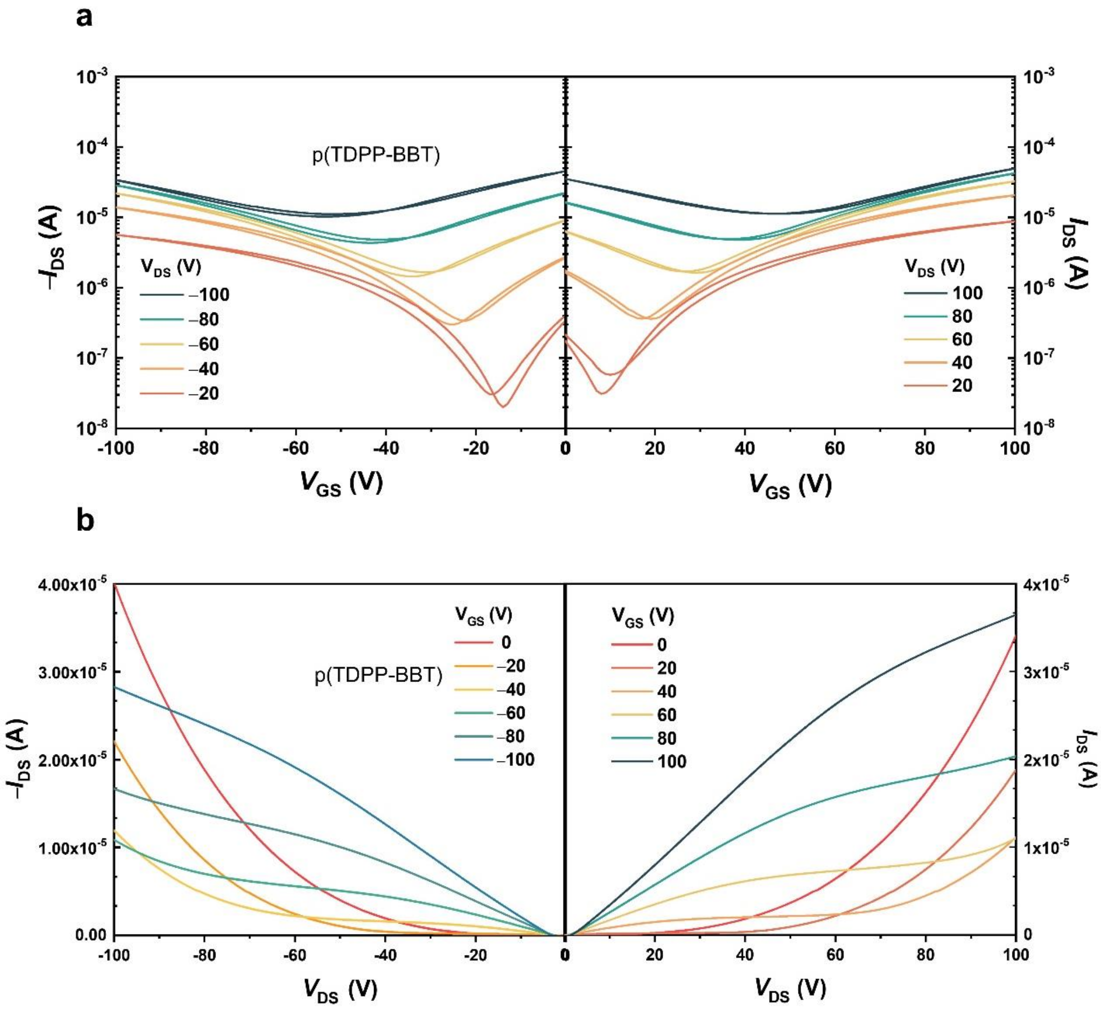


**Supplementary Figure 27. a,** Typical transfer and **b,** output characteristics of p(TDPP-BBT) OFET devices fabricated in glovebox and tested in ambient conditions ($L$ = 10 μm, $W$ = 200 μm). 500 nm thick CYTOP was used as the dielectric layer ($C_i$ = 3.7 nF cm$^{-2}$).

To obtain the saturation mobility, $V_{DS}$ = −100 V for the p-type regime and $V_{DS}$ = +100 V for the n-type regime are used to satisfy $V_{DS} > |V_{GS} - V_{Th}|$. We linearly fitted the slope of the square root of the drain current (Supplementary Figure 28) and used Eq. 5,

$$\mu_{sat} = \left(\frac{\partial\sqrt{I_{DS,sat}}}{\partial(V_G - V_{Th})}\right)^2 \frac{2L}{WC_i} \qquad \text{(Eq. 5)}^{12}$$

to calculate the charge carrier mobilities.

As shown in the following figure (Supplementary Figure 28), typical ambipolar transfer characteristics were obtained. The square root of the drain current shows good linearity at high $V_{GS}$ regime. We did not observe any noticeable non-idealities in our

devices. Ambipolar FET devices usually show large $V_{Th}$ because of the relatively high injection barrier from the gold electrodes for both hole and electron. To precisely calculate the mobility values, we chose the large linear fitting ranges from $|V_{GS}|$ = 70-100 V, except for p(TDPP-BT) working in the n-type regime (Supplementary Figure 28). The LUMO energy level of p(TDPP-BT) is higher, leading to a higher electron injection barrier and larger $V_{Th}$. Thus, we fitted its saturation mobilities from $|V_{GS}|$ = 85-100 V.

We also measured the charge carrier mobilities in the linear regime. As shown in the output curves (Supplementary Fig. 25, 26b, 27b), some of the FET devices of p(TDPP-BT) exhibited lightly larger contact resistances and larger $V_{Th}$. To calculate the mobilities in the linear regime, $V_{DS} = -20$ V for p-type regime and $V_{DS} = +20$ V for n-type regime were used to satisfy $V_{DS} < |V_{GS} - V_{Th}|$. We linearly fitted the slope of the drain current (Supplementary Fig. 29) and used Eq. 6,

$$\mu_{lin} = \frac{\partial I_{DS}}{\partial V_{GS}} \frac{L}{W C_i V_{DS}} \qquad \text{(Eq. 6)}^{12}$$

to calculate the mobilities. Similarly, the fitting range is $|V_{GS}|$ = 70-100 V (Supplementary Fig. 29). For p(TDPP-BT) working in the n-type regime, we fitted its linear mobilities from $|V_{GS}|$ = 85-100 V because of its large $V_{Th}$.

The average saturation and linear mobility values with standard deviations for the three polymers are summarized in Supplementary Table 7. Note that the linear regime mobilities are lower than the saturation regime mobilities, which is common for organic semiconductors[13]. The lower linear mobilities are probably due to the relatively larger contact resistance and higher injection barrier in ambipolar organic semiconductors[14, 15].

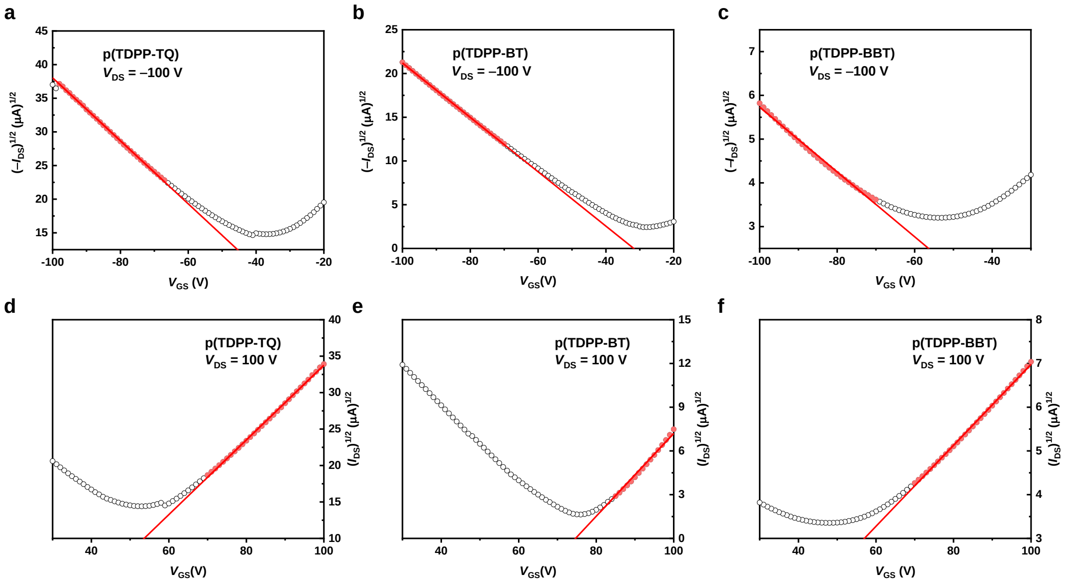


**Supplementary Figure 28.** Fitting ranges of the hole and electron mobilities of **a, d,** p(TDPP-TQ), **b, e,** p(TDPP-BT), and **c, f,** p(TDPP-BBT) in the saturation regime. The red circles are the selected fitting ranges, and the solid red lines are the fitting results. OFET devices ($L$ = 10 μm, $W$ = 200 μm) were fabricated with a dielectric layer of CYTOP around 500 nm thick ($C_i$ = 3.7 nF cm$^{-2}$).

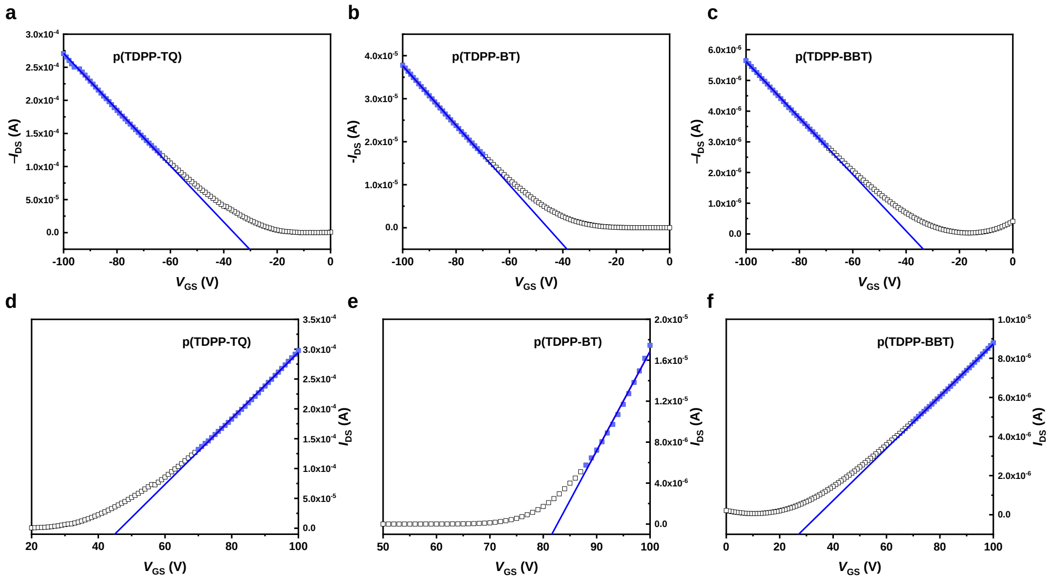


**Supplementary Figure 29.** Fitting ranges of the hole and electron mobilities of **a, d,** p(TDPP-TQ), **b, e,** p(TDPP-BT), and **c, f,** p(TDPP-BBT) in the linear regime. The blue squares are the fitting ranges, and the solid blue lines are the fitting results. OFET devices ($L$ = 10 μm, $W$ = 200 μm) were fabricated with a dielectric layer CYTOP around 500 nm thick ($C_i$ = 3.7 nF cm$^{-2}$).

**Supplementary Table 7.** Extracted hole and electron mobilities of the polymer FET devices operated in saturation and linear regime.

| Polymer | $\mu_{h, saturation}$ ($cm^2\ V^{-1}\ s^{-1}$) | $\mu_{e, saturation}$ ($cm^2\ V^{-1}\ s^{-1}$) | $\mu_{h, linear}$ ($cm^2\ V^{-1}\ s^{-1}$) | $\mu_{e, linear}$ ($cm^2\ V^{-1}\ s^{-1}$) |
|---|---|---|---|---|
| p(TDPP-BT) | 2.77±0.15 | 3.83±0.65 | 0.56±0.10 | 0.75±0.35 |
| p(TDPP-TQ) | 6.16±0.68 | 7.76±0.86 | 3.15±0.39 | 4.05±0.57 |
| p(TDPP-BBT) | 0.25±0.06 | 0.35±0.08 | 0.08±0.01 | 0.12±0.05 |

**Supplementary Table 8.** Charge transport properties of some reported high-spin organic semiconductors.

| | Chemical structure | Ground state | Hole mobility ($cm^2\ V^{-1}\ s^{-1}$) | Electron mobility ($cm^2\ V^{-1}\ s^{-1}$) | References |
|---|---|---|---|---|---|
| 1 | 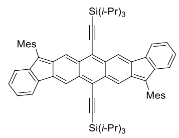 | singlet | $2 \times 10^{-3}$ | $4 \times 10^{-3}$ | [16] |
| 2 | 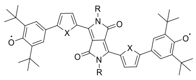 | singlet | / | 0.004 | [17] |
| 3 | 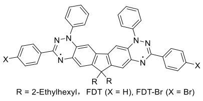 | / | FDT: $1.7 \times 10^{-5}$, FDT-Br: $2.4 \times 10^{-5}$ | / | [18] |
| 4 | 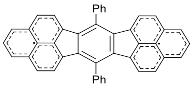 | / | $2.6 \times 10^{-3}$ | $3.2\times10^{-3}$ | [19] |
| 5 | 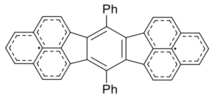 | / | $7.2 \times 10^{-1}$ (single crystal) | / | [20] |

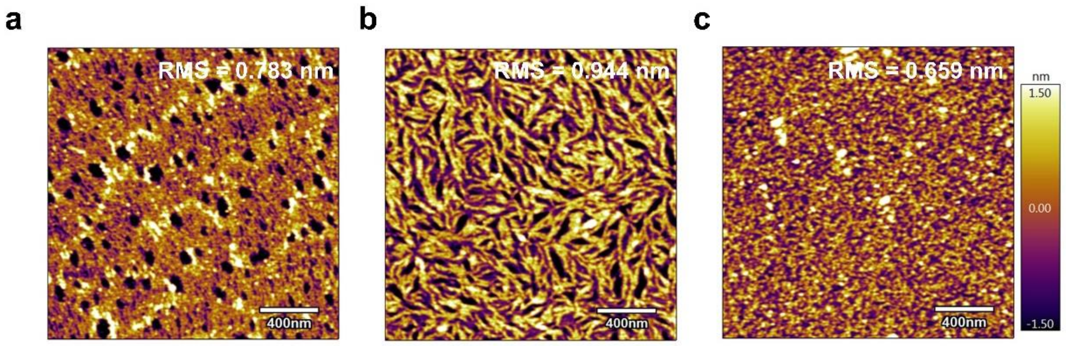


**Supplementary Figure 30.** AFM height images of the polymer films. **a**, p(TDPP-BT) (RMS = 0.783 nm), **b,** p(TDPP-TQ) (RMS = 0.944 nm) and **c,** p(TDPP-BBT) (RMS = 0.659 nm).

**Polymer doping and electrical conductivity measurement.**

**N-doping** used in this work is solution blending. p(TDPP-TQ) was dissolved in 1,2-dichlorobenzene (*o*-DCB) with a concentration of 3 g/L. The p(TDPP-TQ) solution was blended with dopant *N*-DMBI solution as a function of doping ratio at room temperature. After heating the solution at 100 °C for 15 min, the heated solution was spin-coated on the glass substrate at 1500 rpm for 60 s and annealed at 120 °C for 2 h to deposit thin films.

**P-doping** was performed by immersing p(TDPP-TQ) films in a 10 mM $FeCl_3/CH_3NO_2$ solution with varied time. The thick p(TDPP-TQ) films were deposited on the glass substrates by spin-coating its 10 g/L trichloroethylene (TCE) solution at 1000 rpm for 60 s and annealed at 150 °C for 10 min.

All conductvities were collected by four-probe measurements using Keithley 4200 SCS parameter analyzer. The thickness of the films was determined by AFM or by a surface profiler.

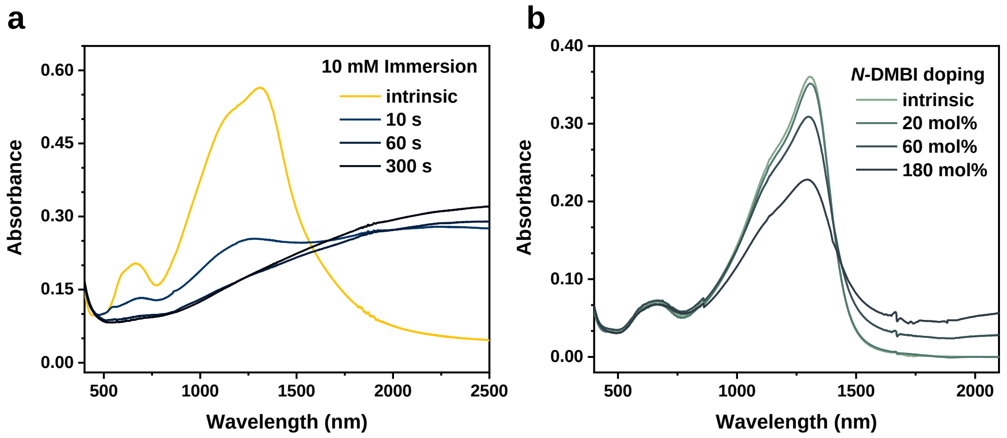


**Supplementary Figure 31.** UV-vis-NIR absorption spectra of doped p(TDPP-TQ) films; **a,** p-doped by immersing in 10 mM $FeCl_3$ solution, and **b,** n-doped by blending with various amount of *N*-DMBI.

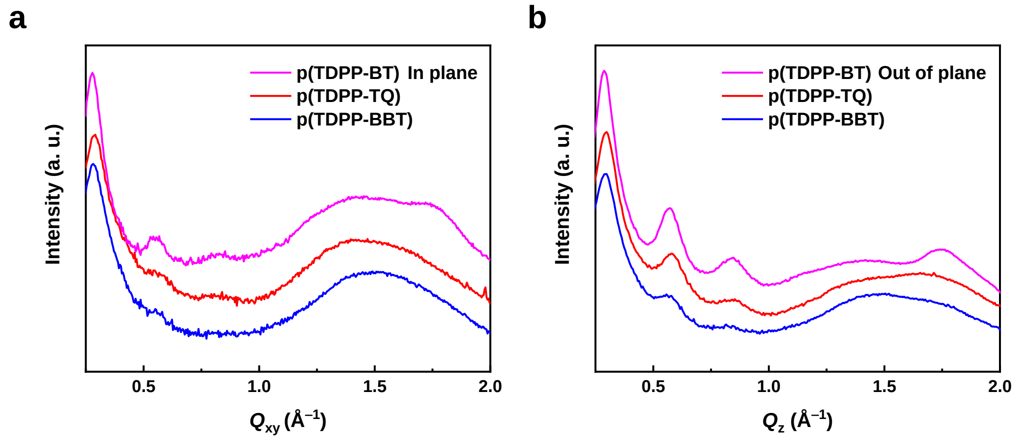


**Supplementary Figure 32. a,** In-plane and **b,** out-of-plane GIWAXS plots of the three polymers.

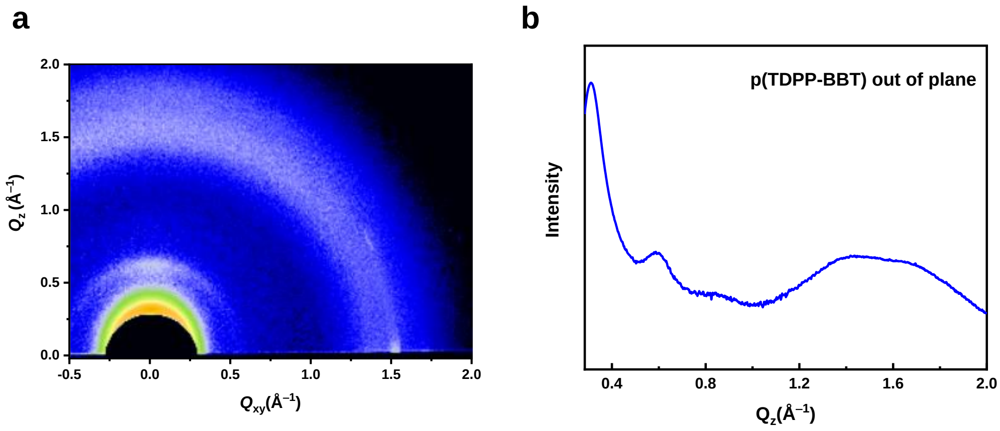


**Supplementary Figure 33. a,** 2D-grazing incidence wide-angle X-ray scattering

(GIWAXS) pattern of p(TDPP-BBT) measured in Shanghai Synchrotron Radiation Facility (SSRF). **b,** out-of-plane GIWAXS plots of p(TDPP-BBT) from the 2D GIWAXS pattern.

**Supplementary Table 9.** The *q*-spacing values, the FWHM of (100) and (010), lamellar distance and π-π distance from the GIWAXS measurement.

| Polymer | (100) [Å$^{-1}$] | FWHM of (100) [Å$^{-1}$] | (200) [Å$^{-1}$] | Lamellar distance [Å] | (010) [Å$^{-1}$] | FWHM of (010) [Å$^{-1}$] | π-π distance [Å] |
|---|---|---|---|---|---|---|---|
| p(TDPP-BT) | 0.29 | 0.05 | 0.57 | 21.67 | 1.76 | 0.21 | 3.57 |
| p(TDPP-TQ) | 0.30 | 0.06 | 0.58 | 20.94 | 1.70 | 0.40 | 3.69 |
| p(TDPP-BBT) | 0.31 | 0.05 | 0.60 | 20.27 | 1.70 | 0.66 | 3.69 |

GIWAXS of p(TDPP-BT) and p(TDPP-TQ) was measured in the University of Southern Mississippi. And GIWAXS of p(TDPP-BBT) was measured on SSRF.

**Air Stability** We measured the stability of n-doped and p-doped p(TDPP-TQ) in air (Supplementary Fig. 34). The doped polymer showed moderate air stability compared with other n-type and p-type polymers. For example, Caironi *et al.*[21] reported the air stability of n-doped PNDIT2. The electrical conductivities decreased to 10% within 100 min. For our polymer p(TDPP-TQ), the stability is better, and its conductivities remain 40% within 100 min (Supplementary Fig. 34a). Pei *et al.*[22] reported the best air-stable n-type polymer LPPVs with a LUMO energy level of −4.49 eV. LPPV-1 film showed only a 39% loss of conductivity after 76 days of exposure to ambient conditions, which is largely due to its extremely low LUMO energy levels.

p(TDPP-TQ) can also be effectively p-doped by $FeCl_3$. The p-type electrical conductivities remained 70% after 1 h in the ambient conditions (Supplementary Fig. 34b). Brinkmann *et al.*[23] reported the air stability of $FeCl_3$-doped $C_{12}$-PBTTT. They showed that the electrical conductivities remained around 20% after 1 h exposure to air.

The decrease of p-doped electrical conductivity is probably due to the formation of $Fe(OH)_2$ through the de-doping reaction by $FeCl_4^-$ in the presence of water and oxygen[24].

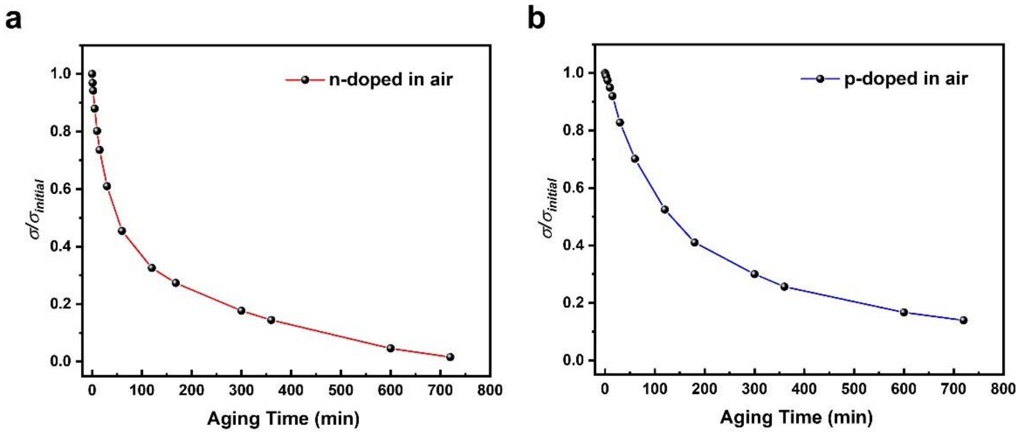


**Supplementary Figure 34.** The air stability of p(TDPP-TQ) film **a,** n-doped with 100 mol% *N*-DMBI, and **b,** p-doped with 10 mM $FeCl_3$. Humidity: 50-60%, Temperature: 293 K.

**Supplementary Note 8. Material synthesis and characterization**

**Materials and General Methods.** All reactions and manipulations were carried out under nitrogen atmosphere or in nitrogen filled glovebox. 3,6-di(thiophen-2-yl)-2,5-dihydropyrrolo[3,4-c]pyrrole-1,4-dione was synthesized according to the literature.[25] $^1$H NMR spectra were recorded on Bruker ARX-400 (400 MHz). All chemical shifts were reported in parts per million (ppm). $^1$H NMR chemical shifts were referenced to $CDCl_3$ (7.26 ppm).

**Monomer synthesis and purification:**

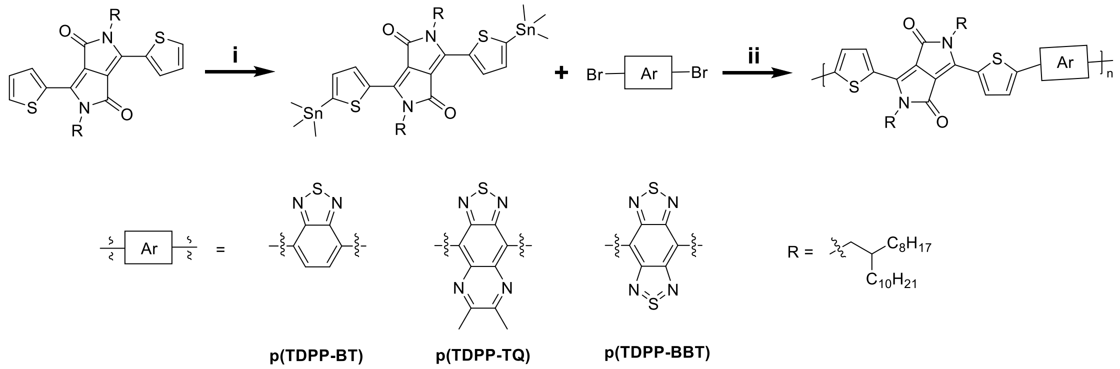


Synthetic route of three polymers. Reactions conditions: (i) −78 °C, LDA, b, $Me_3SnCl$;

(ii) microwave, (ii) $Pd(PPh_3)_4$, o-xylenes, 120 °C for 5 mins, 140 °C for 5 mins, 170 °C for 30 mins.

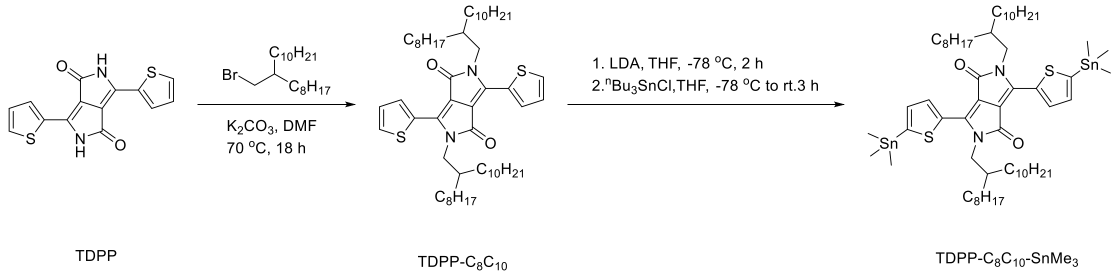


TDPP-$C_8C_{10}$: Under nitrogen atomosphere, 3,6-di(thiophen-2-yl)-2,5-dihydropyrrolo[3,4-c]pyrrole-1,4-dione (TDPP) (3.0 g, 10.0 mmol), potassium carbonate (5.5 g, 40.0 mmol), and dry DMF were added to a 250 mL round-bottom flask,. The mixture was heated to 120 °C for 30 mins. After the mixture was cooled to room temperature, 9-(2-bromoethyl)nonadecane (18.8 g, 50.0 mmol) was added in one portion. The reaction was then stirred at 80 °C for 18 h. After the mixture was cooled to room temperature, it was poured to cool water, then extracted with $CH_2Cl_2$. Then the extract was washed thoroughly with saturated saltwater and dried with $Na_2SO_4$. The crude product was purified by column chromatography on silica using $CH_2Cl_2$/petroleum ether as eluent to get TDPP-$C_8C_{10}$ as dark puper solid (4.5 g, 52%). $^1H$ NMR (400 MHz, $CDCl_3$) δ 8.87, 8.86, 7.62, 7.61, 7.27, 7.26, 7.25, 4.01, 1.90, 1.57, 1.21, 0.87, 0.85.

TDPP-$C_8C_{10}$-$SnMe_3$: Under nitrogen atomosphere, 2.4 M *n*-BuLi (1.5 mL, 3.48 mmol) was addded dropwise to 15 mL THF solution of diisopropylamine (352.2 mg, 3.48 mmol) at −78 °C. The reaction was slowly warmed to room temperature and reacted for 30 mins. Then the mixture was added to 70 mL THF solution of TDPP-$C_8C_{10}$ (1.16 mmol, 1.0g) at −78 °C, the reaction was stirred at −78 °C for 2 h. Then $Me_3SnCl$ (4.06 mmol, 857.8 mg) was added dropwise at −78 °C and keep for 30 mins. The reaction was slowly warmed to room temperature and reacted for 3 h. After the reaction was finished, the solution was poured to cool water and extracted with $CH_2Cl_2$. The extract was washed thoroughly with saturated saltwater and dried with $Na_2SO_4$. The crude

product was purified by recrystal from ethanol/$CH_2Cl_2$ to get TDPP-$C_8C_{10}$-$SnMe_3$ as dark red solid (826.2 mg, 60%). $^1$H NMR (400 MHz, $CDCl_3$) δ 8.99, 8.98, 7.32, 7.31, 4.05, 4.03, 1.91, 1.21, 0.88, 0.87, 0.85, 0.83, 0.43.

**Polymerization and polymer purification:**

p(TDPP-BT): 45.7 mg, yield 92 %. GPC analysis $M_n$ = 42.0 kg/mol, PDI = 4.90 (against PS standard). $^1$H NMR (500 MHz, 1,1,2,2-tetrachloroethane-$d_2$, 363 K, ppm): δ 9.22, 8.07, 4.14, 2.5-0.5. Elemental Anal. Calcd for $(C_{60}H_{88}N_4O_2S_3)_n$: C, 72.53; H, 8.93; N, 5.64, Found: C, 71.80; H, 8.98; N, 5.12.

p(TDPP-TQ): 47.6 mg, yield 90 %. GPC analysis $M_n$ = 16.1 kg/mol, PDI = 3.72 (against PS standard). $^1$H NMR (500 MHz, 1,1,2,2-tetrachloroethane-$d_2$, 363 K, ppm): δ 9.76, 9.48, 9.18, 3.16, 2.5-0.5. Elemental Anal. Calcd for $(C_{64}H_{92}N_6O_2S_3)_n$: C, 71.60; H, 8.64; N, 7.83, Found: C, 69.70; H, 8.41; N, 7.36.

p(TDPP-BBT): 43.3 mg, yield 82 %. GPC analysis $M_n$ = 21.0 kg/mol, PDI = 3.1 (against PS standard). $^1$H NMR (500 MHz, 1,1,2,2-tetrachloroethane-$d_2$, 363 K, ppm): δ 9.39, 7.71, 7.48, 2.0-0.5. Elemental Anal. Calcd for $(C_{60}H_{86}N_6O_2S_4)_n$: C, 68.53; H, 8.24; N, 7.99, Found: C, 67.83; H, 8.19; N, 7.47.

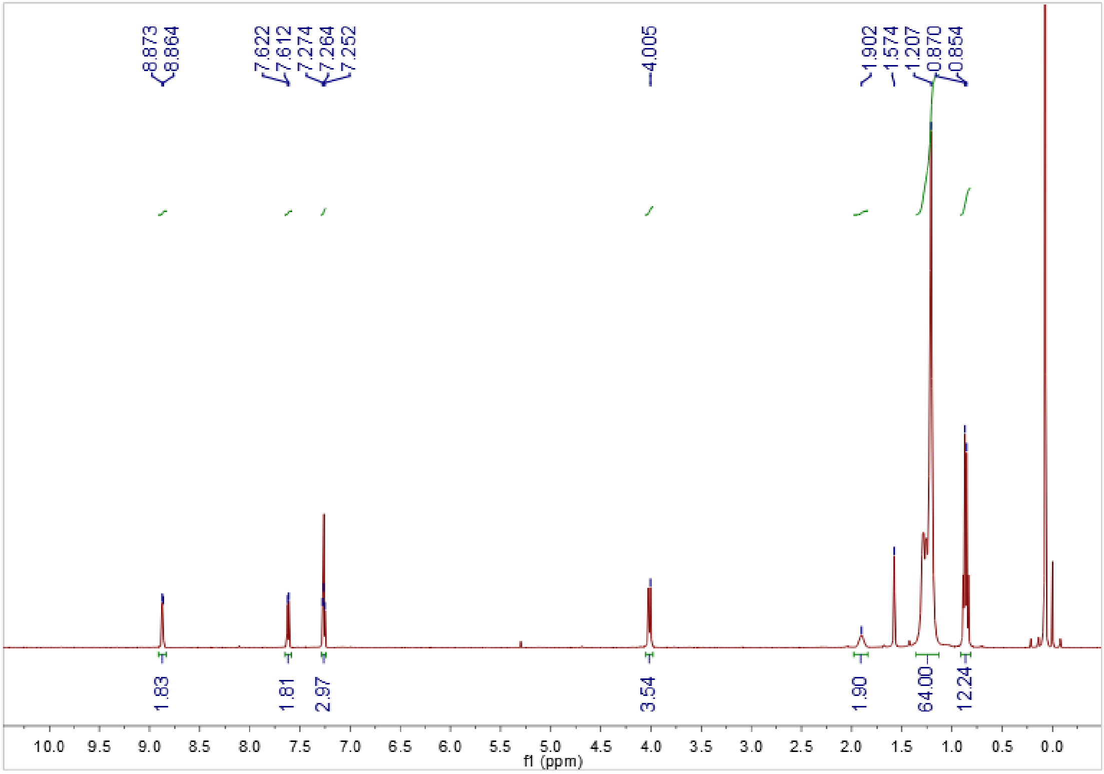


**Supplementary Figure 35.** $^{1}H$ NMR spectrum (500 MHz) of TDPP-$C_8C_{10}$ in $CDCl_3$ at 298 K.

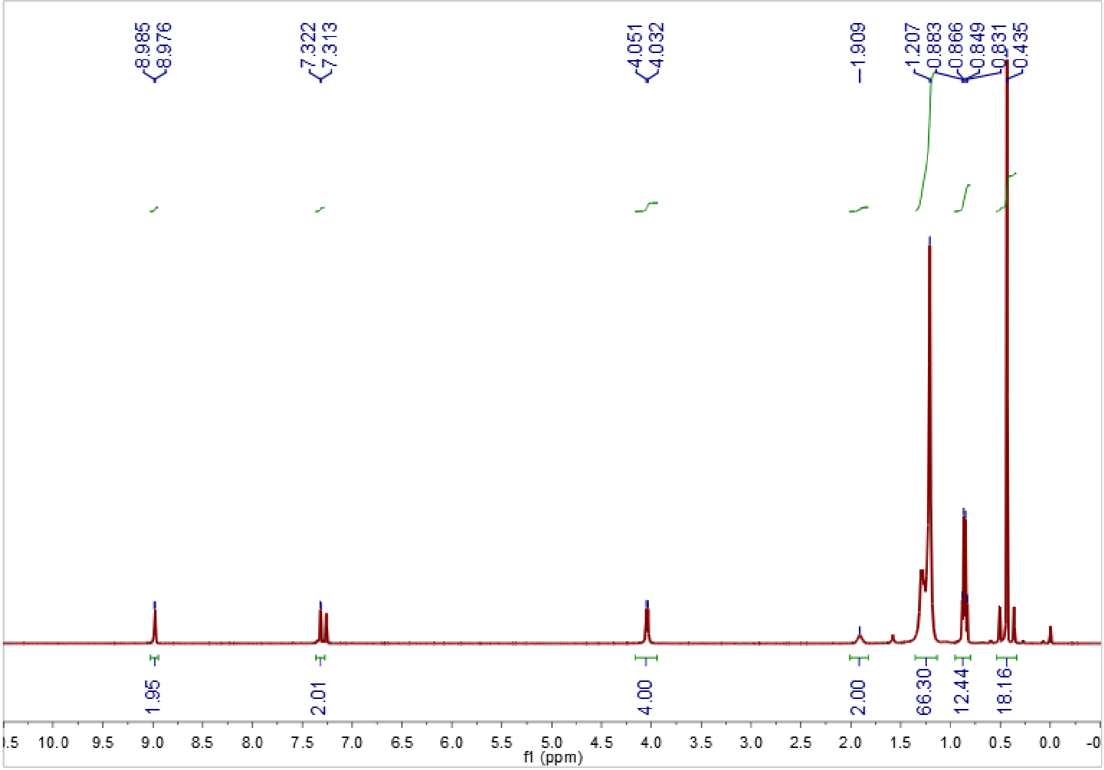

**Supplementary Figure 36.** $^{1}H$ NMR spectrum (500 MHz) of TDPP-$C_8C_{10}$-$SnMe_3$ in $CDCl_3$ at 298 K

## Supplementary References:


1. Gregory SA, *et al.* Quantifying charge carrier localization in chemically doped semiconducting polymers. *Nat. Mater.* **20**, 1414-1421 (2021).
2. Eaton GR, Eaton, S. S., Barr, D. P., Weber, R. T. *Quantitative EPR*. Springer-Verlag/Wien (2010).
3. Oliver K. *Molecular Magnetism*. VCH-Verlag, Weinheim, New York (1993).
4. Goldberg IB, Crowe HR, Newman PR, Heeger AJ, Macdiarmid AG. Electron-spin Resonance of Polyacetylene and $AsF_5$-doped Polyacetylene. *J. Chem. Phys.* **70**, 1132-1136 (1979).
5. Tanaka H, Hirate M, Watanabe S, Kuroda S-i. Microscopic signature of metallic state in semicrystalline conjugated polymers doped with fluoroalkylsilane molecules. *Adv. Mater.* **26**, 2376-2383 (2014).
6. Kang K, *et al.* 2D coherent charge transport in highly ordered conducting polymers doped by solid state diffusion. *Nat. Mater.* **15**, 896-902 (2016).
7. Pal SK, Itkis ME, Tham FS, Reed RW, Oakley RT, Haddon RC. Resonating valence-bond ground state in a phenalenyl-based neutral radical conductor. *Science* **309**, 281-284 (2005).
8. Pal SK, *et al.* Phenalenyl-based neutral radical molecular conductors: Substituent effects on solid-state structures and properties. *J. Am. Chem. Soc.* **129**, 7163-7174 (2007).
9. Ji X, *et al.* Pauli paramagnetism of stable analogues of pernigraniline salt featuring ladder-type constitution. *J. Am. Chem. Soc.* **142**, 641-648 (2020).
10. Haddon RC, Ramirez AP, Glarum SH. Electron-electron Interactions in Organic Superconductors. *Adv. Mater.* **6**, 316-321 (1994).
11. Small D, Zaitsev V, Jung YS, Rosokha SV, Head-Gordon M, Kochi JK. Lntermolecular,pi-to-pi bonding between stacked aromatic dyads. Experimental and theoretical binding energies and near-IR optical transitions for phenalenyl

radical/radical versus radical/cation dimerizations. *J. Am. Chem. Soc.* **126**, 13850-13858 (2004).
12. Zaumseil J, Sirringhaus H. Electron and Ambipolar Transport in Organic Field-Effect Transistors. *Chem. Rev.* **107**, 1296-1323 (2007).
13. He M, *et al.* Donor–Acceptor Conjugated Polymers Based on Dithieno[3,2-b:3',2'-b']naphtho[1,2-b:5,6-b']dithiophene: Synthesis and Semiconducting Properties. *Macromolecules* **49**, 825-832 (2016).
14. Sirringhaus H. 25th Anniversary Article: Organic Field-Effect Transistors: The Path Beyond Amorphous Silicon. *Adv. Mater.* **26**, 1319-1335 (2014).
15. Kang H, *et al.* Megahertz-class printed high mobility organic thin-film transistors and inverters on plastic using attoliter-scale high-speed gravure-printed sub-5μm gate electrodes. *Org. Electron.* **15**, 3639-3647 (2014).
16. Rudebusch GE, *et al.* Diindeno-fusion of an anthracene as a design strategy for stable organic biradicals. *Nat. Chem.* **8**, 753-759 (2016).
17. Wang W, *et al.* Fine-tuning the diradical character of molecular systems via the heteroatom effect. *Chem. Commun.* **56**, 1405-1408 (2020).
18. Zhang Y, Zheng Y, Zhou H, Miao M-S, Wudl F, Thuc-Quyen N. Temperature tunable self-doping in stable diradicaloid thin-film devices. *Adv. Mater.* **27**, 7412-7419 (2015).
19. Kubo T, *et al.* Synthesis, intermolecular interaction, and semiconductive behavior of a delocalized singlet biradical hydrocarbon. *Angew. Chem. Int. Ed.* **44**, 6564-6568 (2005).
20. Koike H, *et al.* Stable delocalized singlet biradical hydrocarbon for organic field-effect transistors. *Adv. Funct. Mater.* **26**, 277-283 (2016).
21. Nava D, *et al.* Drastic Improvement of Air Stability in an n-Type Doped Naphthalene-Diimide Polymer by Thionation. *ACS Appl. Energy Mater.* **1**, 4626-4634 (2018).
22. Lu Y, *et al.* Rigid Coplanar Polymers for Stable n-Type Polymer Thermoelectrics. *Angew. Chem. Int. Ed.* **58**, 11390-11394 (2019).
23. Vijayakumar V, *et al.* Bringing Conducting Polymers to High Order: Toward Conductivities beyond $10^5$ S $cm^{-1}$ and Thermoelectric Power Factors of 2 mW $m^{-1}$ $K^{-2}$. *Adv. Energy Mater.* **9**, 1900266 (2019).
24. Lee S, Paine DC, Gleason KK. Heavily Doped poly(3,4-ethylenedioxythiophene) Thin Films with High Carrier Mobility Deposited Using Oxidative CVD: Conductivity Stability and Carrier Transport. *Adv. Funct. Mater.* **24**, 7187-7196 (2014).
25. Huo L, *et al.* Bandgap and Molecular Level Control of the Low-Bandgap Polymers Based on 3,6-Dithiophen-2-yl-2,5-dihydropyrrolo[3,4-c]pyrrole-1,4-dione toward Highly Efficient Polymer Solar Cells. *Macromolecules* **42**, 6564-6571 (2009).
